# Supplementary material for: Engineering triangular carbon quantum dots with unprecedented narrow bandwidth emission for multicolored LEDs
Source: Nat Commun. 2018 Jun 8;9:2249. doi: 10.1038/s41467-018-04635-5 (PMC5993800; doi:10.1038/s41467-018-04635-5)
Supplement: Supplementary file 1 — Supplementary Information [file 41467_2018_4635_MOESM1_ESM.pdf]

Supplementary Information

Engineering Triangular Carbon Quantum Dots with Unprecedented Narrow Bandwidth Emission for Multicolored LEDs

Fanglong Yuan, Ting Yuan, Laizhi Sui, Zhibin Wang, Zifan Xi, Yunchao Li, Xiaohong Li, Louzhen Fan, Zhan'ao Tan,* Anmin Chen, Mingxing Jin* and Shihe Yang**

Supplementary Figures

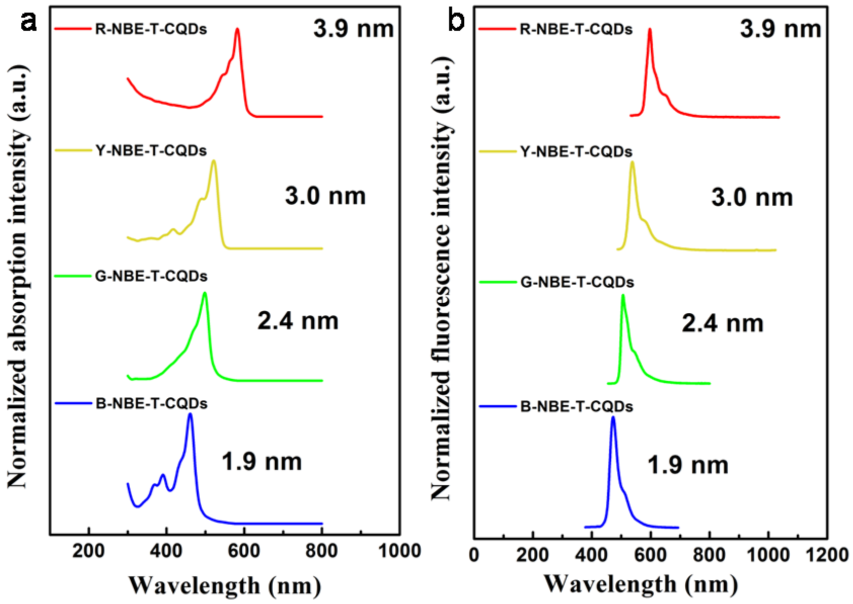

Supplementary Figure 1. Normalized UV-vis absorption (a) and PL (b) spectra of B-, G-, Y-, and R-NBE-T-CQDs.

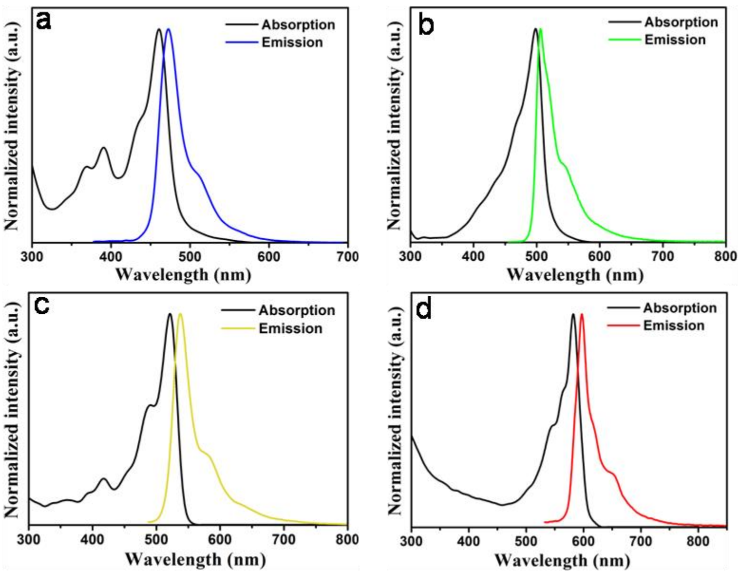

Supplementary Figure 2. Normalized UV-vis absorption and PL spectra of B- (a), G- (b), Y- (c), and R-NBE-T-CQDs (d).

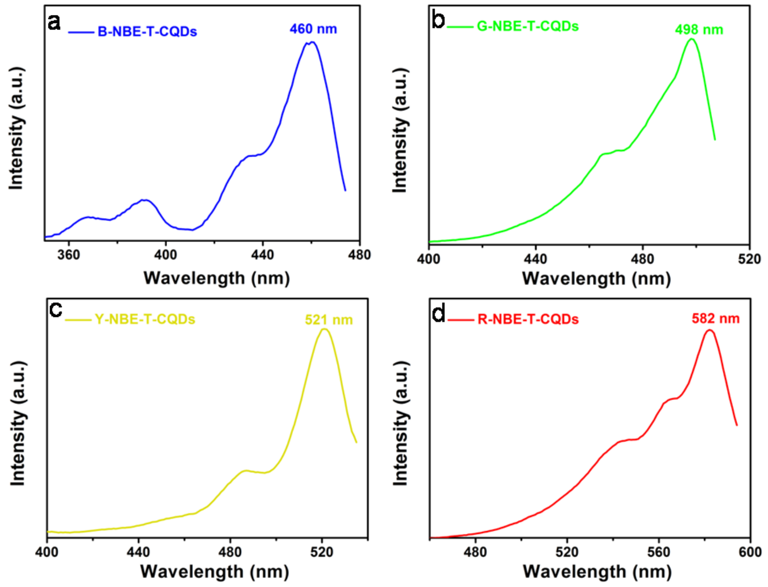

Supplementary Figure 3. The FL excitation spectra of B- (a), G- (b), Y- (c), and R-NBE-T-CQDs (d).

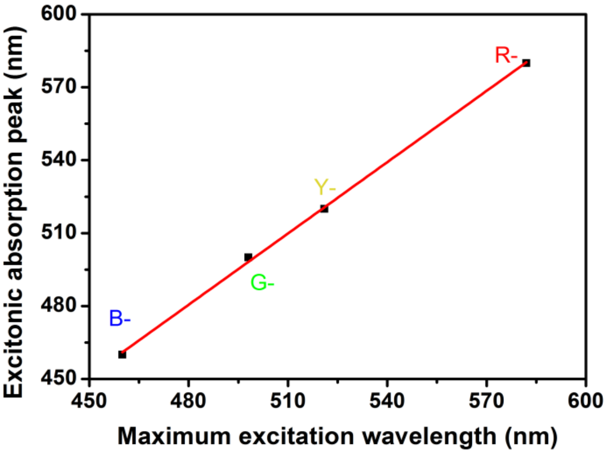

Supplementary Figure 4. The relationship between the maximum excitation wavelength of the PL emissionand the corresponding excitonic absorption peak.

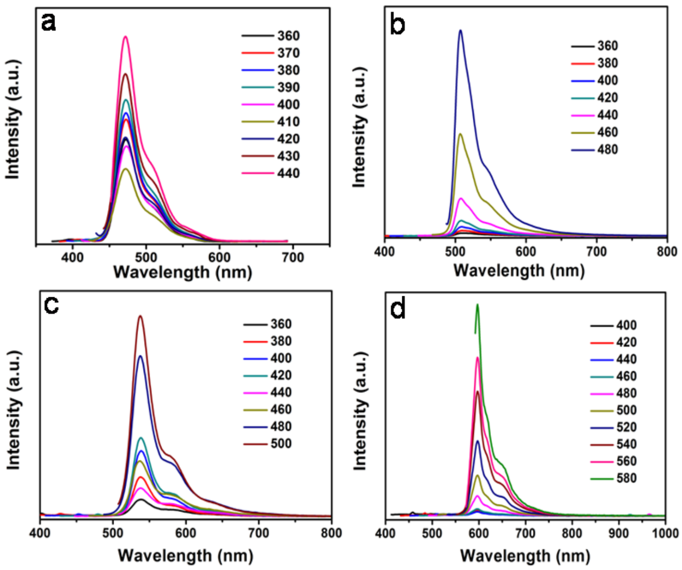

Supplementary Figure 5. PL spectra of B- (a), G- (b), Y- (c), and R-NBE-T-CQDs (d) excited at different wavelengths.

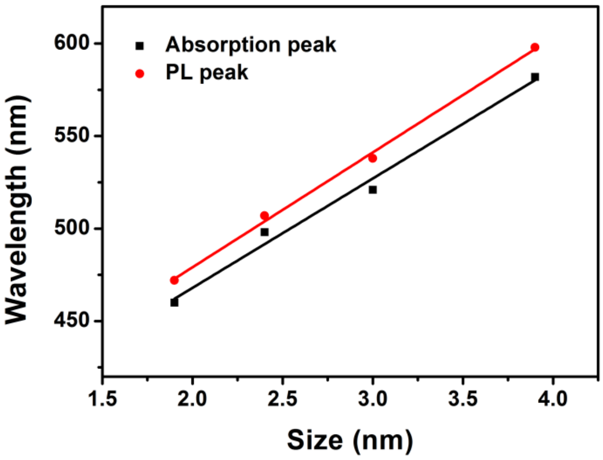

Supplementary Figure 6. Dependence of the PL and the first excitonic absorption peak wavelength on the size of NBE-T-CQDs.

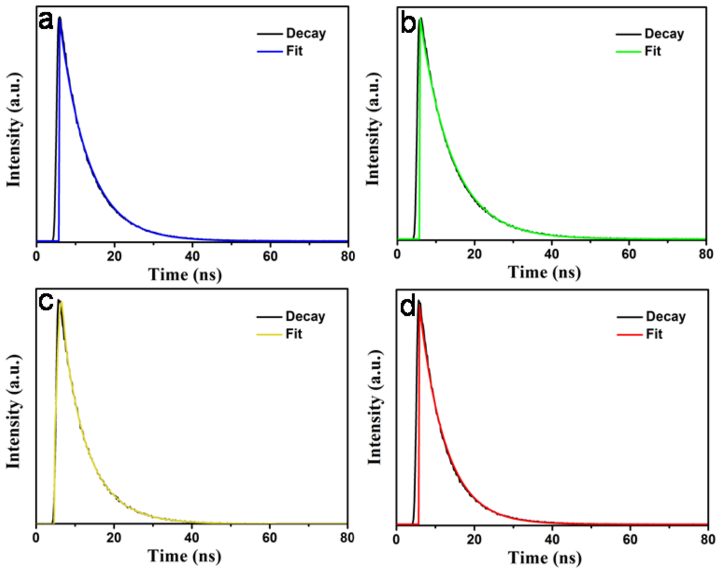

Supplementary Figure 7. PL decay traces of B- (a), G- (b), Y- (c), and R-NBE-T-CQDs (d) excited at 460, 490, 520 and 580 nm with corresponding PL peaks at 472, 507, 538 and 598 nm, respectively.

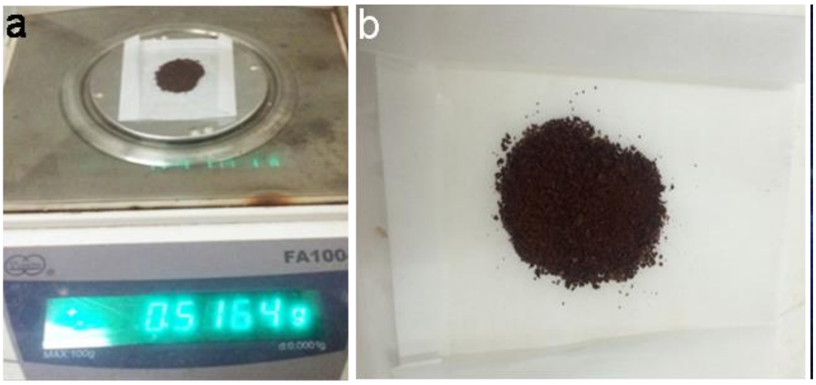

Supplementary Figure 8. Optical photographs of the G-NBE-T-CQDs powder (about 516 mg) under daylight obtained from large-scale solvothermal synthesis followed by purification via silica column chromatography.

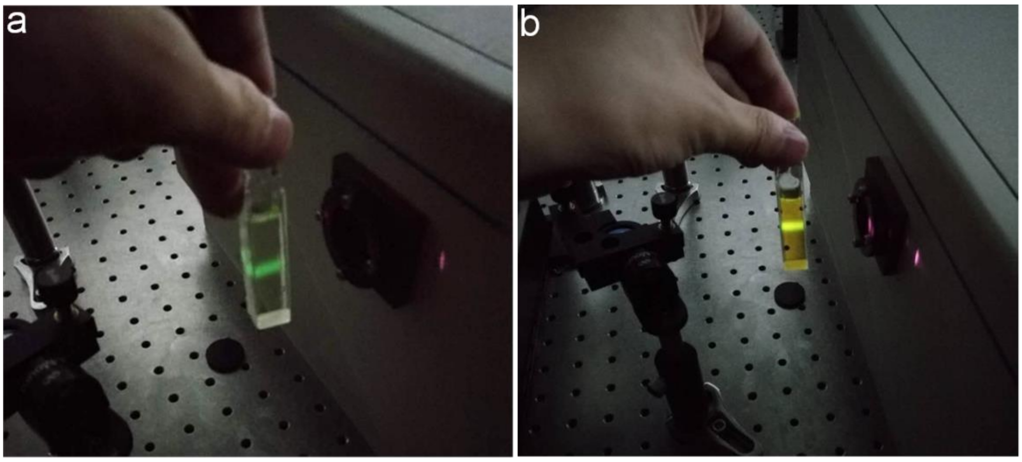

Supplementary Figure 9. Two-photon fluorescence images of diluted (a) and concentrated (b) G-NBE-T-CQDs ethanol solutionsunder 800 nm femtosecond laser excitation.

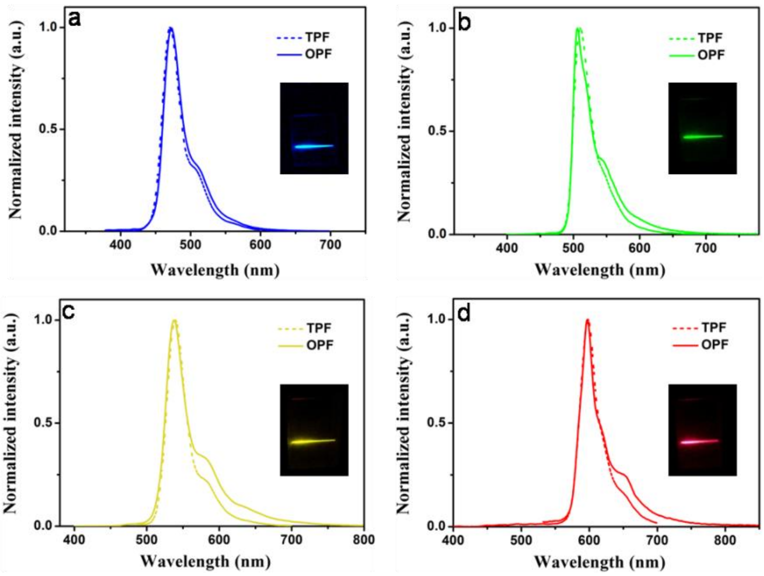

Supplementary Figure 10. Normalized one-photon fluorescence (OPF, solid line) and two-photon fluorescence (TPF, short dash line) spectra of B- (a), G- (b), Y- (c), and R-NBE-T-CQDs (d) under 880 nm femtosecond laser excitation. The insets are the TPF images from blue to red of NBE-T-CQDs under 880 nm femtosecond laser excitation.

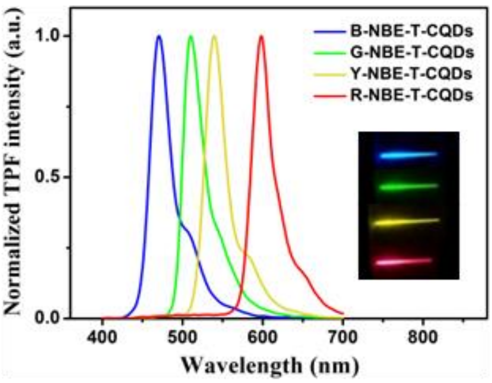

Supplementary Figure 11. Normalized TPF spectra of NBE-T-CQDs under 880 nm femtosecond laser excitation (Insets are the TPF images from blue to red).

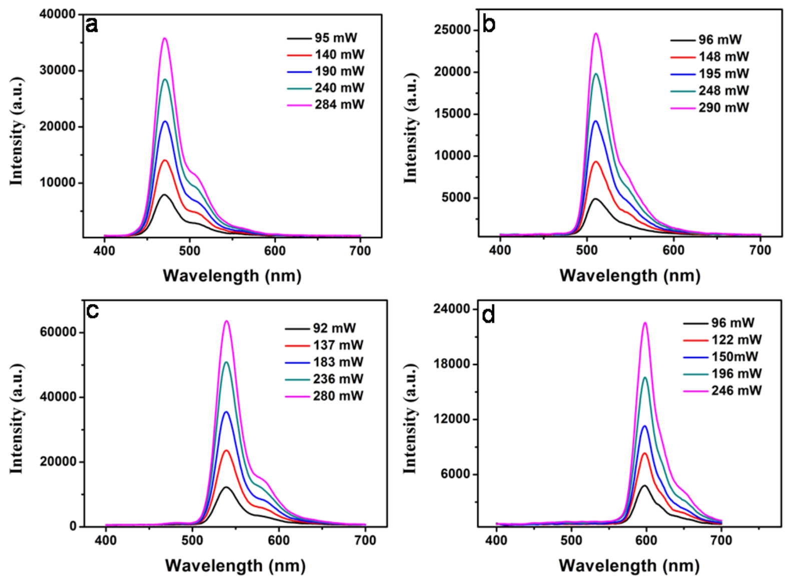

Supplementary Figure 12. TPF spectra of B- (a), G- (b), Y- (c), and R-NBE-T-CQDs (d) under 880 nm femtosecond laser excitation with different laser powers.

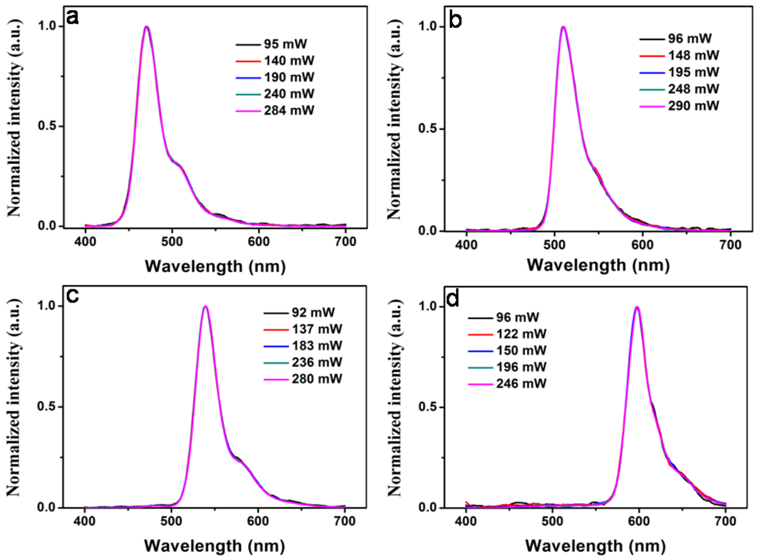

Supplementary Figure 13. Normalized TPF spectra of B- (a), G- (b), Y- (c), and R-NBE-T-CQDs (d) under 880 nm femtosecond laser excitation with different laser powers.

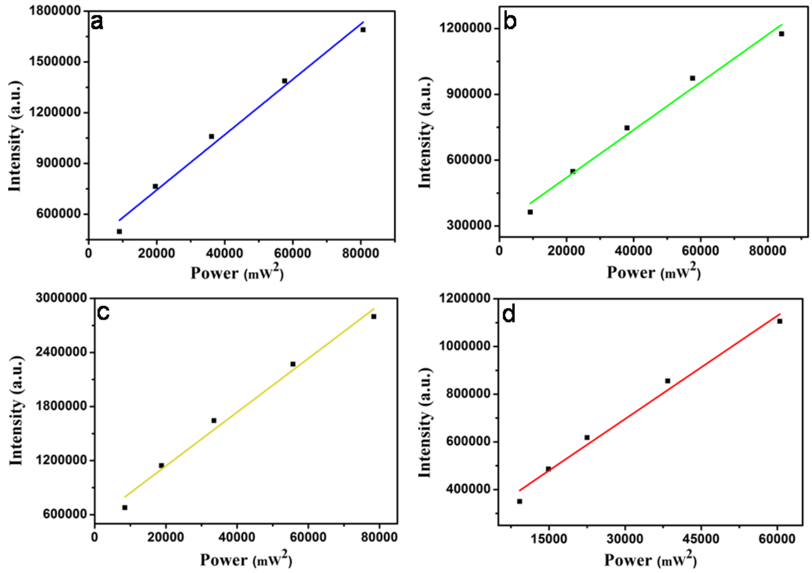

Supplementary Figure 14. Quadratic dependenceof the integrated fluorescence intensity of B- (a), G- (b), Y- (c), and R-NBE-T-CQDs (d) under 880 nm femtosecond laser excitation on the laser power.

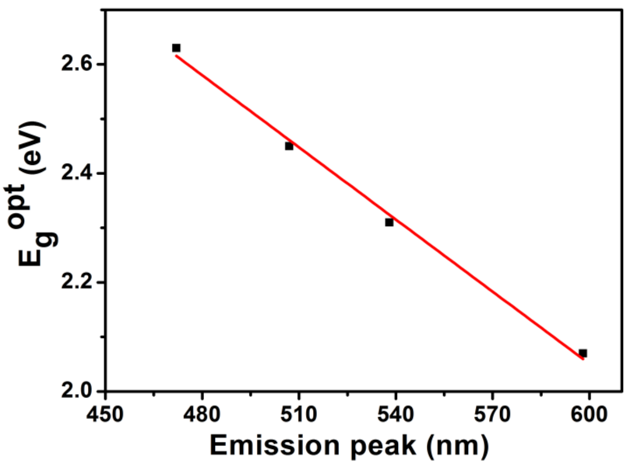

Supplementary Figure 15. The relationship between the bandgap energies and the corresponding excitonic emission peak wavelengths of NBE-T-CQDs.

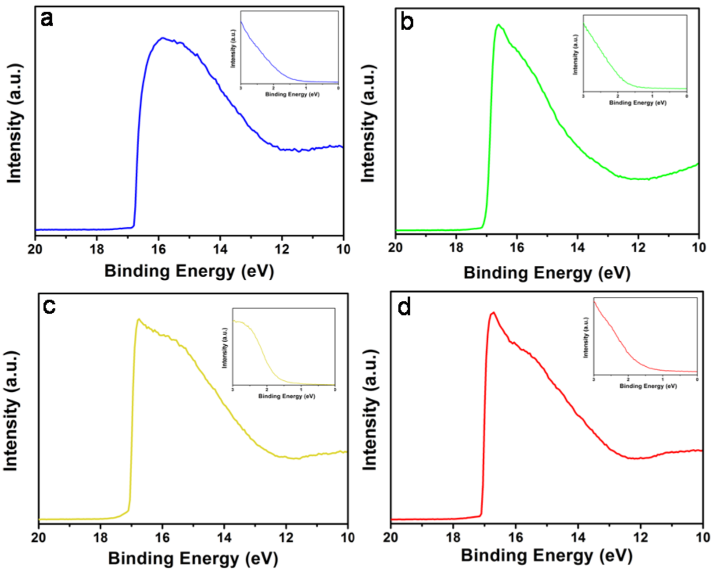

Supplementary Figure 16. Ultraviolet photoelectron spectroscopy (UPS) data of B- (a), G- (b), Y- (c), and R-NBE-T-CQDs (d).

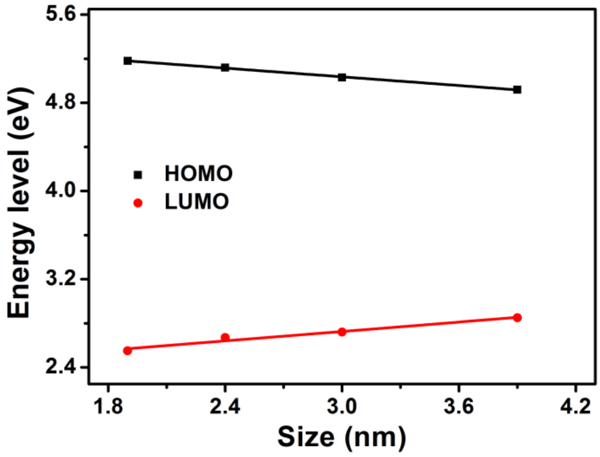

Supplementary Figure 17. The HOMO and LUMO energy levels as a function of the size of NBE-T-CQDs.

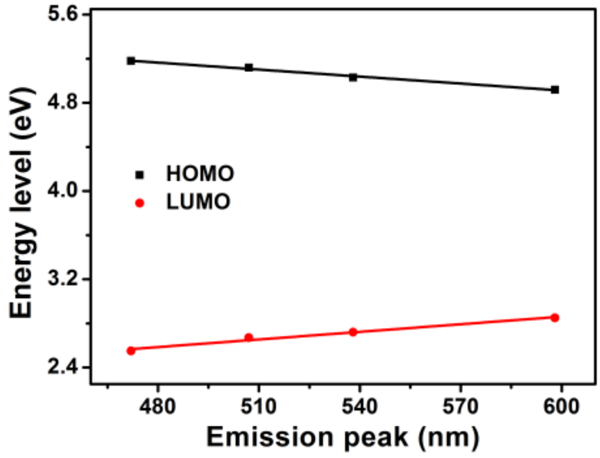

Supplementary Figure 18. The HOMO and LUMO energy levels as a function of the emission peaks of NBE-T-CQDs.

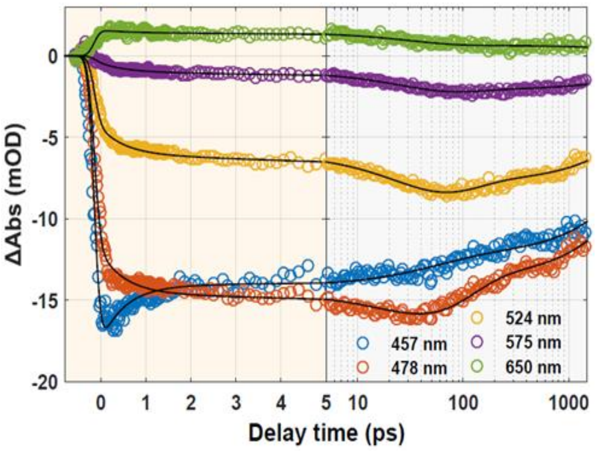

Supplementary Figure 19. The kinetic traces at different probe wavelengths.

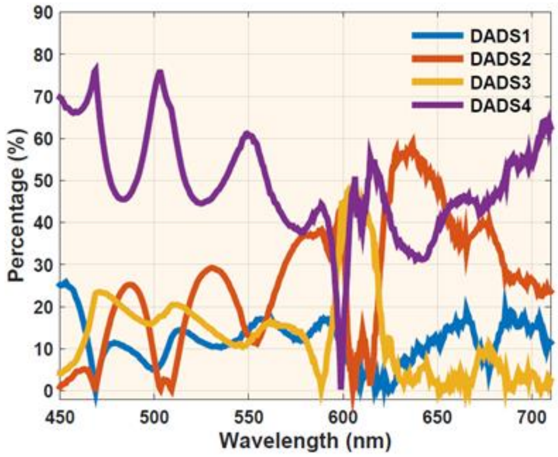

Supplementary Figure 20. The percentages of the four decay channels in the overall dynamics within the wavelength range constructed according to DADS in Figure 2d.

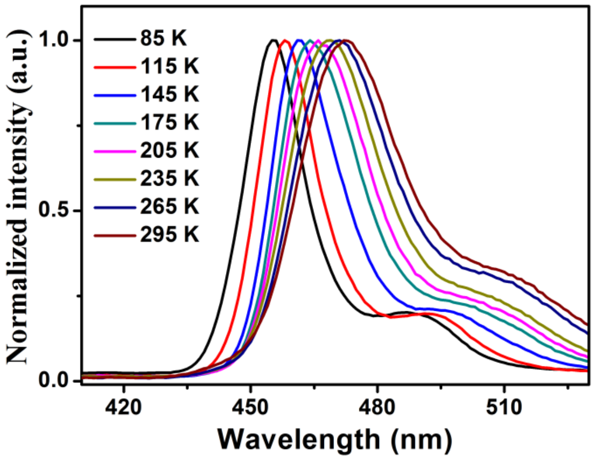

Supplementary Figure 21. Temperature-dependent PL spectra of B-NBE-T-CQDs (85-295 K) excited at 400 nm.

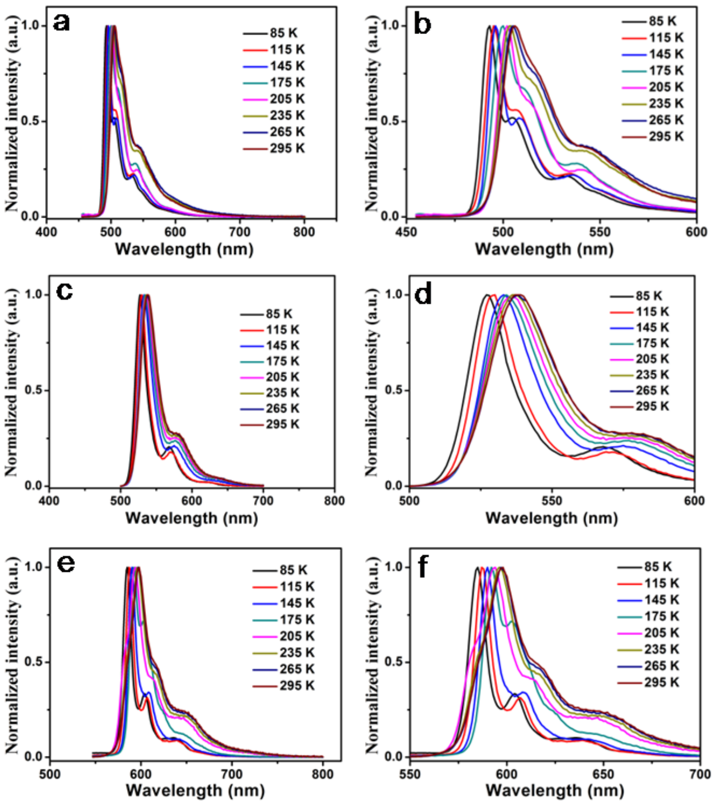

Supplementary Figure 22. Temperature-dependent PL spectra of G- (a, b), Y- (c, d), and R-NBE-T-CQDs (e, f) (85-295 K).

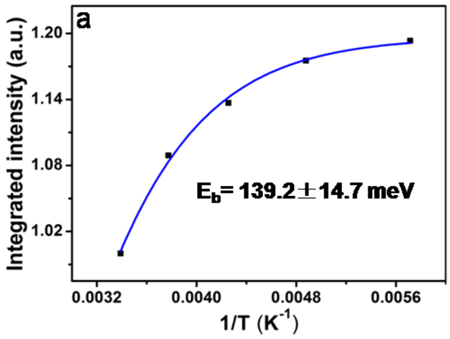

b

	A	B	C	D
1	Model	NewFunction4 (User)		
2	Equation	y = a/(1+b*exp(-c*x))		
3	Reduced Chi-Sqr	3.33031E-5		
4	Adj. R-Square	0.99447		
5			Value	Standard Error
6		a	1.19684	0.00664
7	B	b	46.15524	26.03689
8		c	1613.46706	170.71443

Supplementary Figure 23. The plots of integrated PL emission intensity of B-NBE-T-CQDs as a function of temperature (175-295 K) (a) and the corresponding fitting parameters (b).

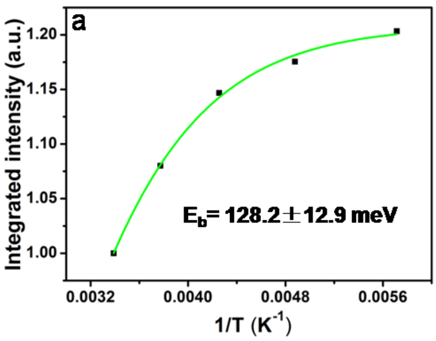

b

	A	B	C	D
1	Model	NewFunction4 (User)		
2	Equation	y = a/(1+b*exp(-c*x))		
3	Reduced Chi-Sqr	3.206E-5		
4	Adj. R-Square	0.9952		
5			Value	Standard Error
6		a	1.2084	0.00718
7	B	b	32.07304	15.81638
8		c	1485.87674	149.9934

Supplementary Figure 24. The plots of integrated PL emission intensity of G-NBE-T-CQDs as a function of temperature (175-295 K) (a) and the corresponding fitting parameters (b).

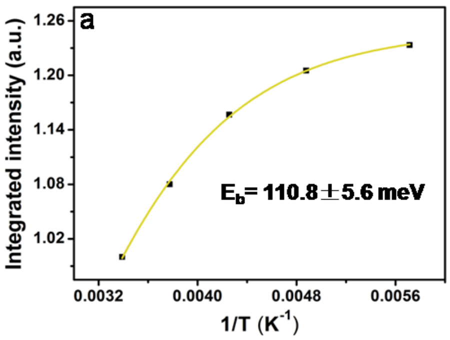

b

	A	B	C	D
1	Model	NewFunction4 (User)		
2	Equation	y = a/(1+b*exp(-c*x))		
3	Reduced Chi-Sqr	9.76439E-6		
4	Adj. R-Square	0.99893		
5			Value	Standard Error
6		a	1.24984	0.00485
7	B	b	19.56008	4.10973
8		c	1284.45303	64.76521

Supplementary Figure 25. The plots of integrated PL emission intensity of Y-NBE-T-CQDs as a function of temperature (175-295 K) (a) and the corresponding fitting parameters (b).

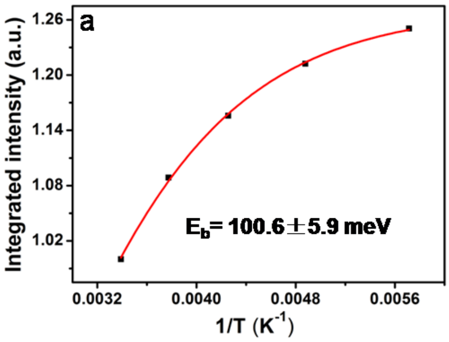

b

	A	B	C	D
1	Model	NewFunction4 (User)		
2	Equation	y = a/(1+b*exp(-c*x))		
3	Reduced Chi-Sqr	1.33106E-5		
4	Adj. R-Square	0.99867		
5			Value	Standard Error
6	B	a	1.27157	0.00657
7		b	14.03882	3.08512
8		c	1166.22649	68.68521

Supplementary Figure 26. The plots of integrated PL emission intensity of R-NBE-T-CQDs as a function of temperature (175-295 K) (a) and the corresponding fitting parameters (b).

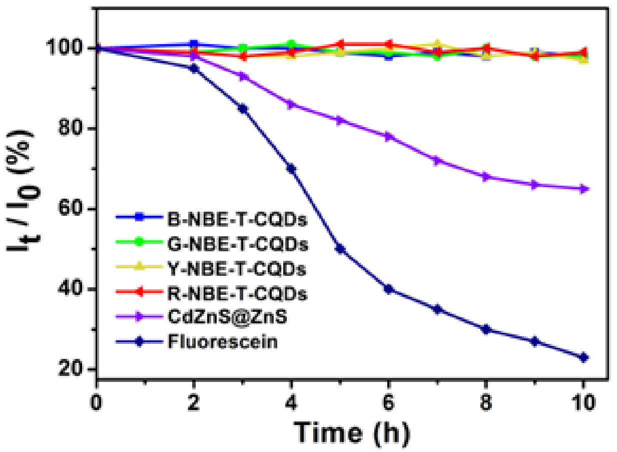

Supplementary Figure 27. The photostability comparison between NBE-T-CQDs and conventional core-shell semiconductor QDs such as CdZnS@ZnS and organic dyes such as fluorescein under continuous radiation with a UV lamp for 10 h.

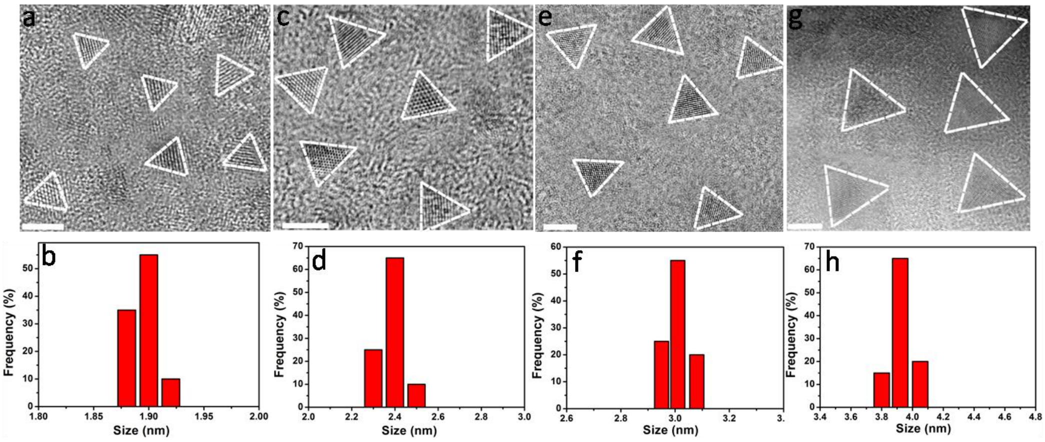

Supplementary Figure 28. TEM images and the corresponding size distribution of B- (a,b), G- (c,d), Y- (e,f), and R-NBE-T-CQDs (g,h). Scale bar, 2 nm. (The triangular projections are highlighted by white contour lines).

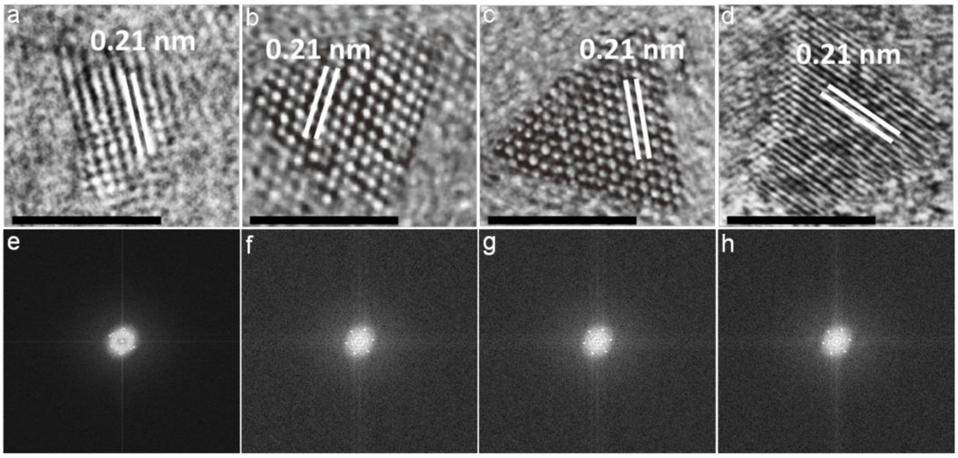

Supplementary Figure 29. The HRTEM images and corresponding fast Fourier transform (FFT) patterns of B- (a, e), G- (b, f), Y- (c, g), and R-NBE-T-CQDs (d, h). Scale bar, 2 nm.

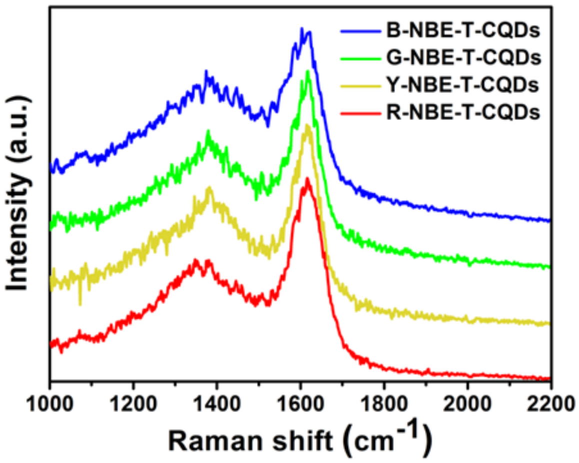

Supplementary Figure 30. Raman spectra of B-, G-, Y-, and R- NBE-T-CQDs excited at 633 nm.

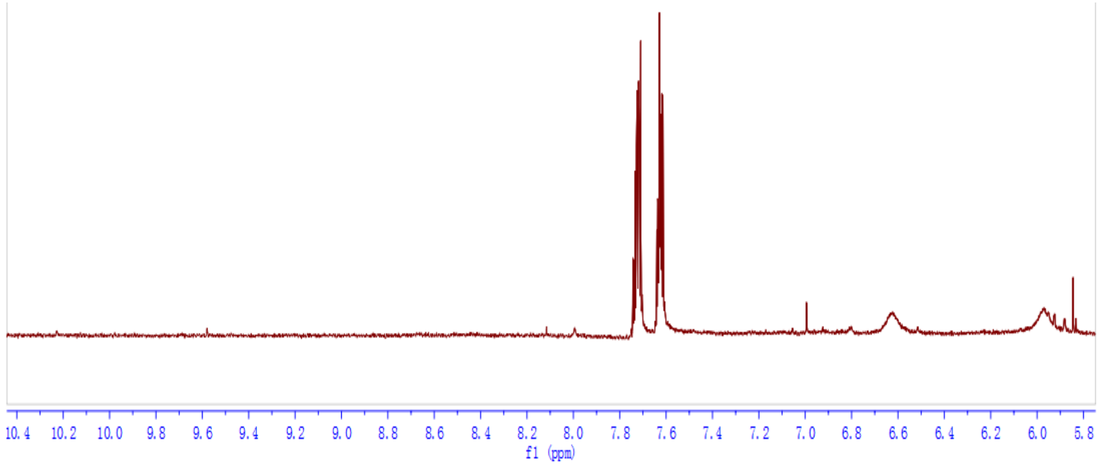

Supplementary Figure 31. ^{1}H-NMR spectra of G-NBE-T-CQDs in acetone-d6.

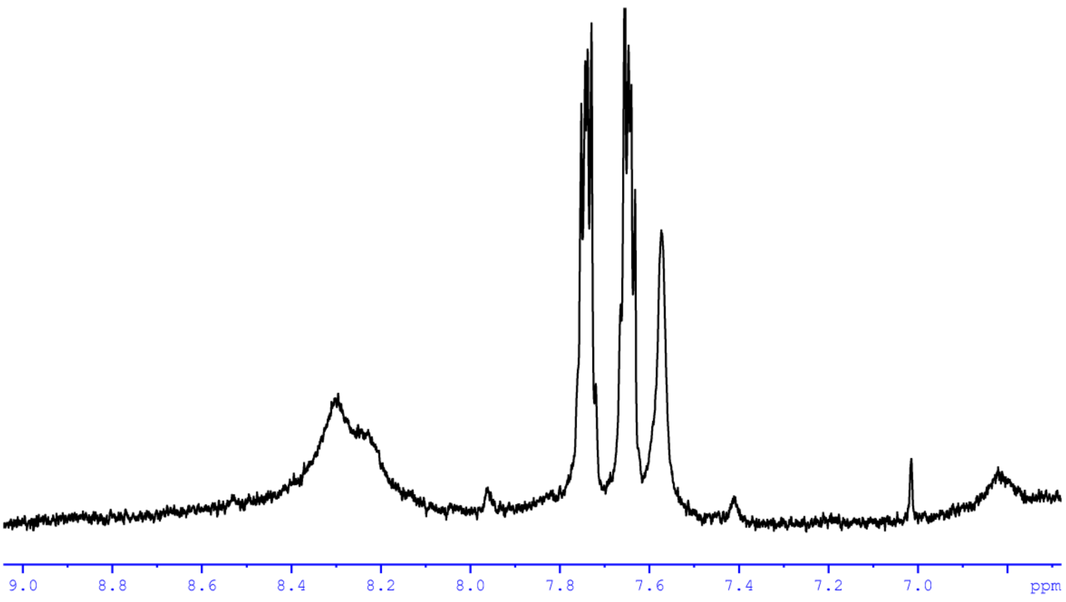

Supplementary Figure 32. ^{1}H-NMR spectra of Y-NBE-T-CQDs in acetone-d6.

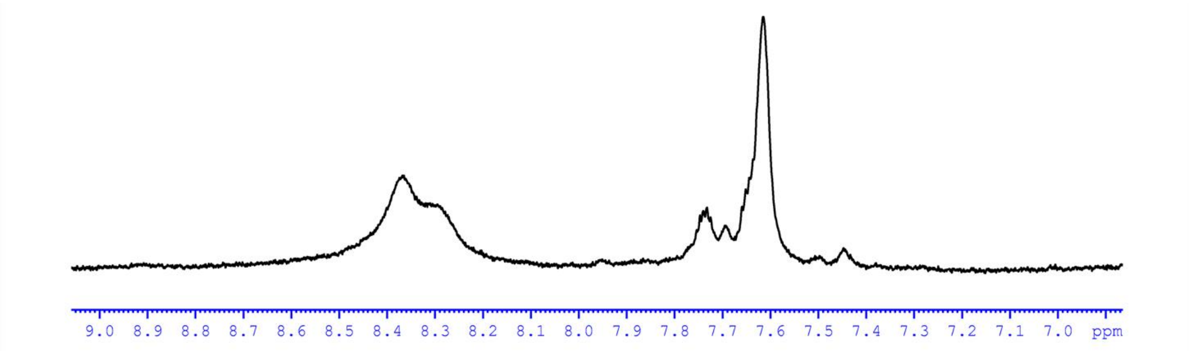

Supplementary Figure 33. ^{1}H-NMR spectra of R-NBE-T-CQDs in acetone-d6.

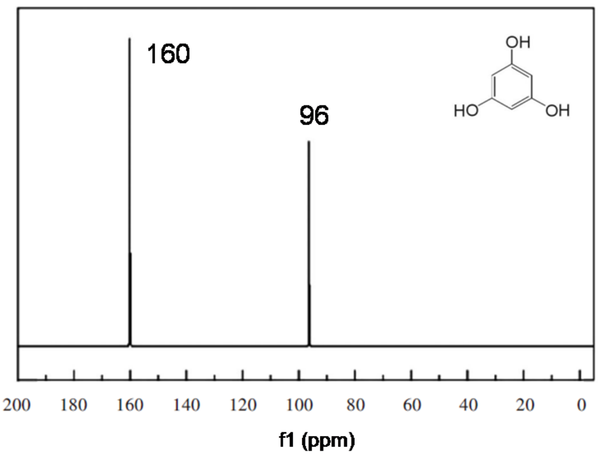

Supplementary Figure 34. ^{13}C-NMR spectra of phloroglucinol in acetone-d6.

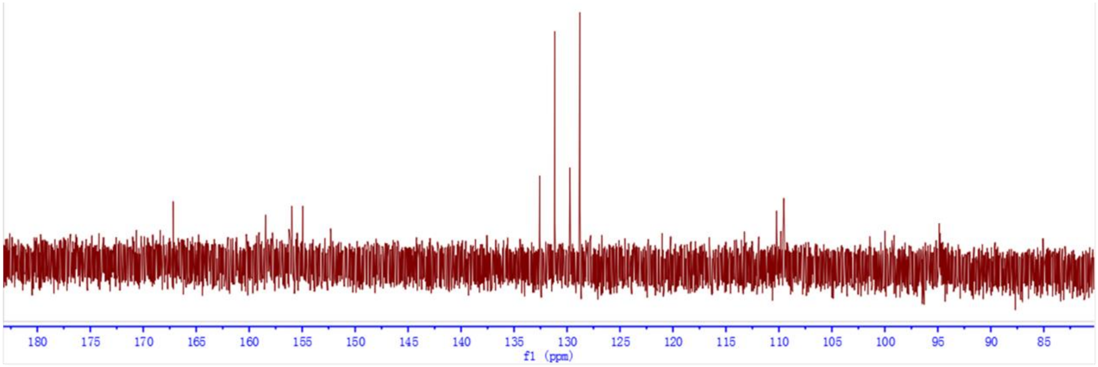

Supplementary Figure 35. ^{13}C-NMR spectra of G-NBE-T-CQDs in methanol-d4.

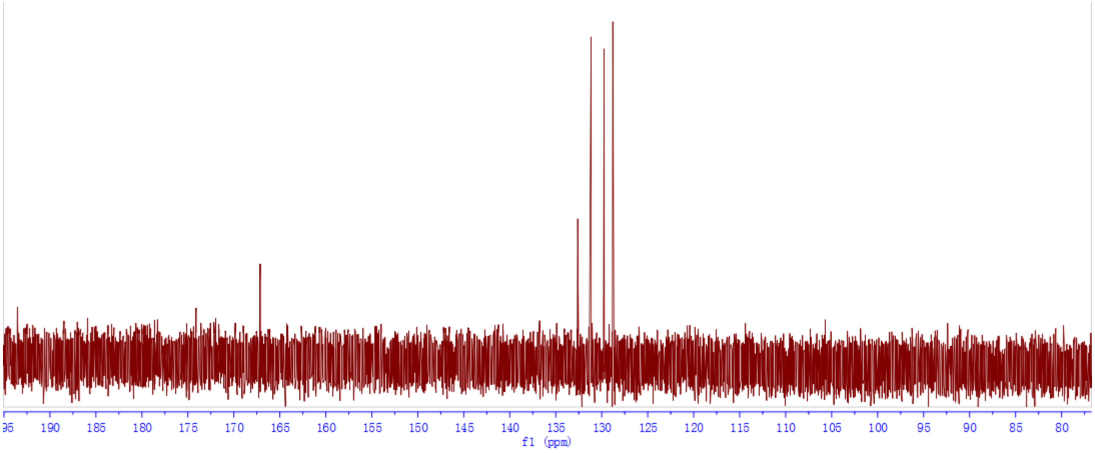

Supplementary Figure 36. ^{13}C-NMR spectra of Y-NBE-T-CQDs in methanol-d4.

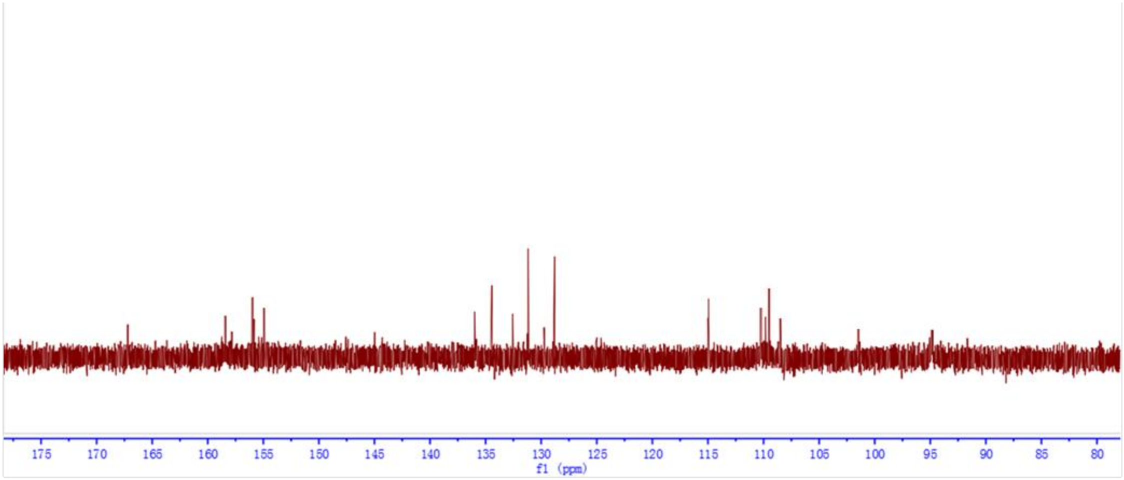

Supplementary Figure 37. ^{13}C-NMR spectra of R-NBE-T-CQDs in methanol-d4.

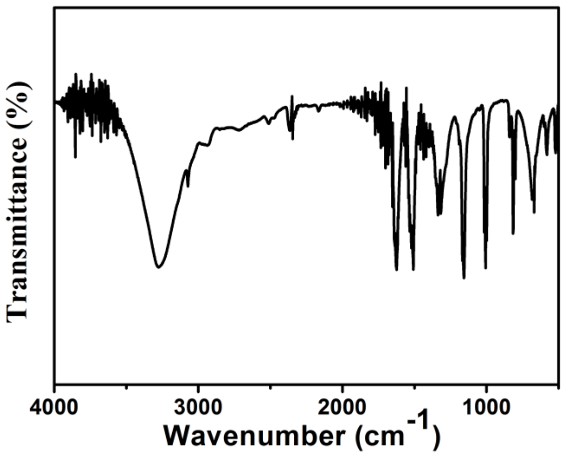

Supplementary Figure 38. FT-IR spectra of phloroglucinol.

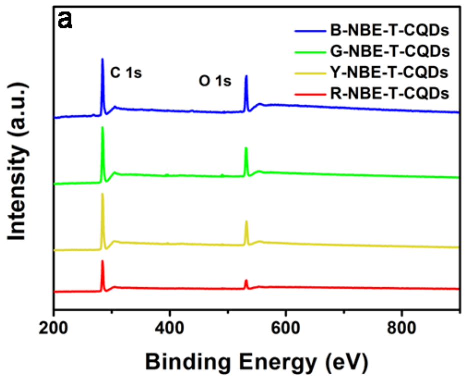

b

Sample	C (%)	O (%)
B-NBE-T-CQDs	78.54	21.46
G-NBE-T-CQDs	82.22	17.78
Y-NBE-T-CQDs	84.46	15.54
R-NBE-T-CQDs	88.45	11.55

Supplementary Figure 39. XPS spectra(a) and the resulting analysis result on the relative contents of C and O atoms (b) of B-, G-, Y-, and R-NBE-T-CQDs.

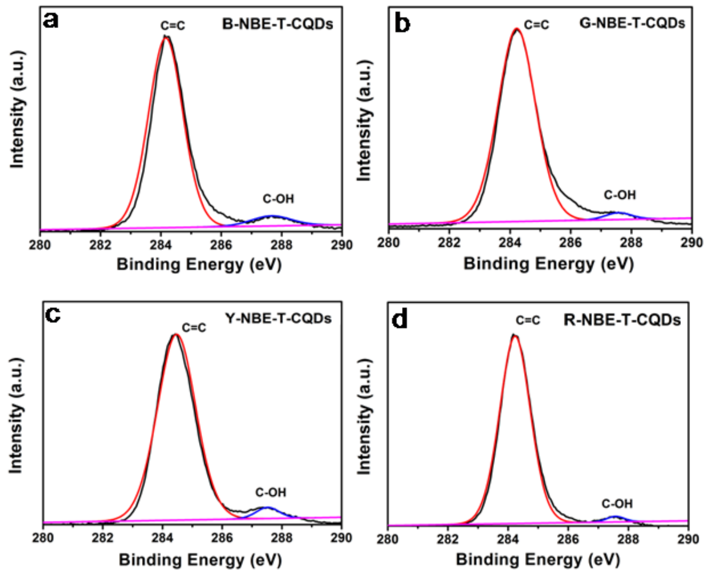

Supplementary Figure 40. High-resolution C1s XPS spectra of B- (a), G- (b), Y- (c), and R-NBE-T-CQDs (d).

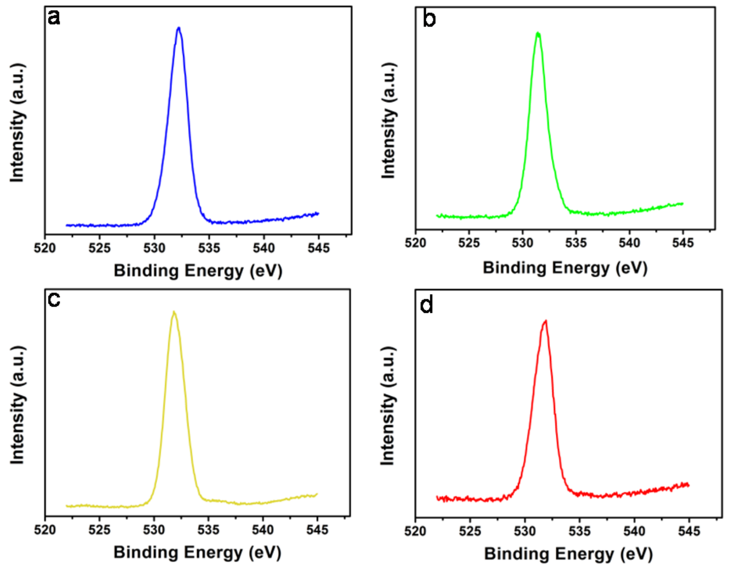

Supplementary Figure 41. High-resolution O1s XPS spectra of B- (a), G- (b), Y- (c), and R-NBE-T-CQDs (d).

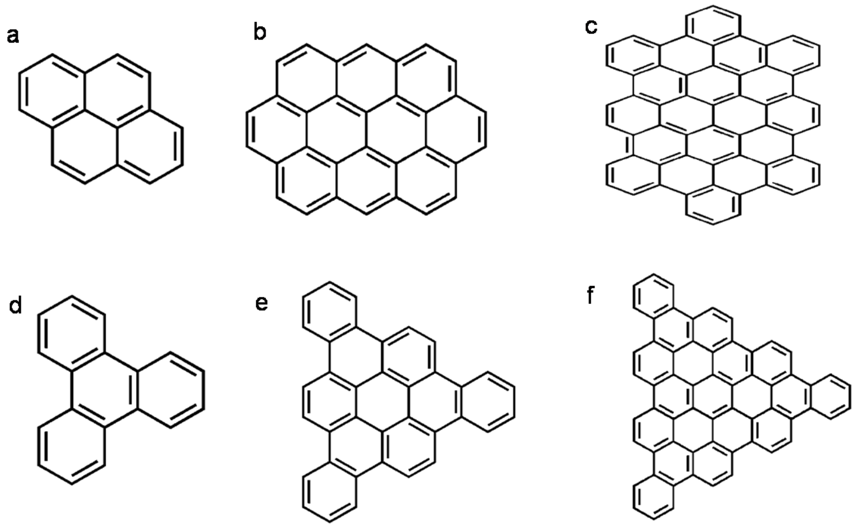

Supplementary Figure 42. The model CQDs withsquare-like structure (named S-CQDs-1 (a), S-CQDs-2 (b), and S-CQDs-3 (c)) and triangular structure (named T-CQDs-1 (d), T-CQDs-2 (e), and T-CQDs-3 (f)).

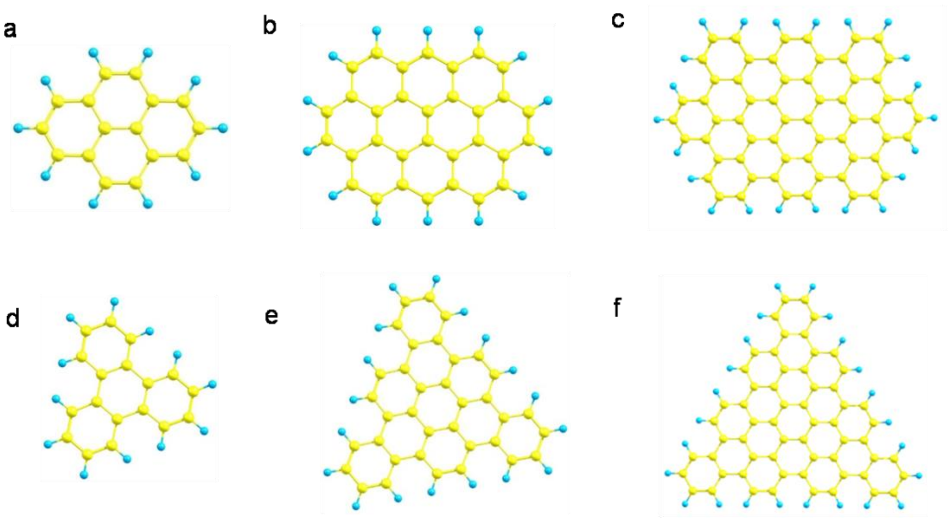

Supplementary Figure 43. The ball-and-stick model of CQDs with square-like structure (named S-CQDs-1 (a), S-CQDs-2 (b), and S-CQDs-3 (c)) and triangular structure (named T-CQDs-1 (d), T-CQDs-2 (e), and T-CQDs-3 (f)).

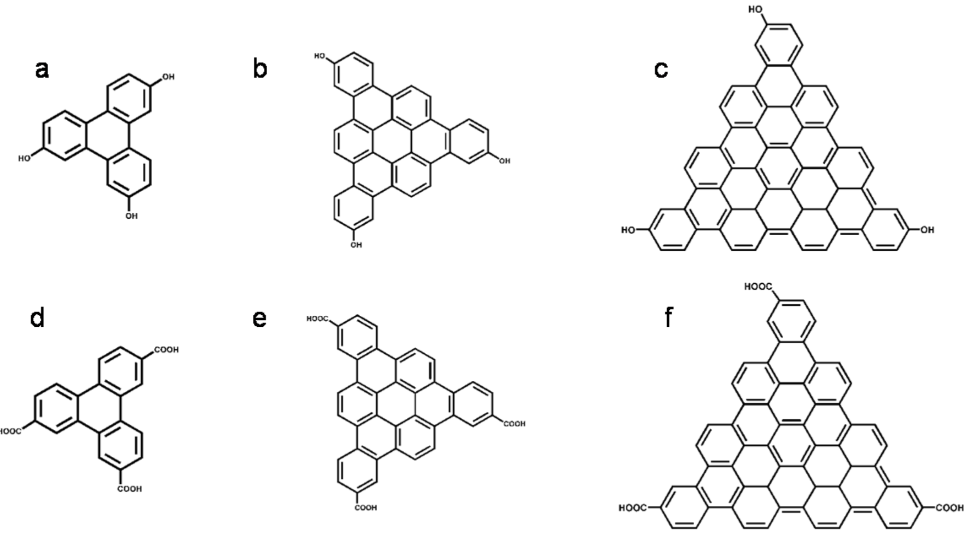

Supplementary Figure 44. The model CQDs with triangular structure functionalized with -OH (named T-CQDs-OH-1 (a), T-CQDs-OH-2 (b), and T-CQDs-OH-3 (c)) and -COOH groups (named T-CQDs-COOH-1 (d), T-CQDs-COOH-2 (e), and T-CQDs-COOH-3 (f)) at edge sites.

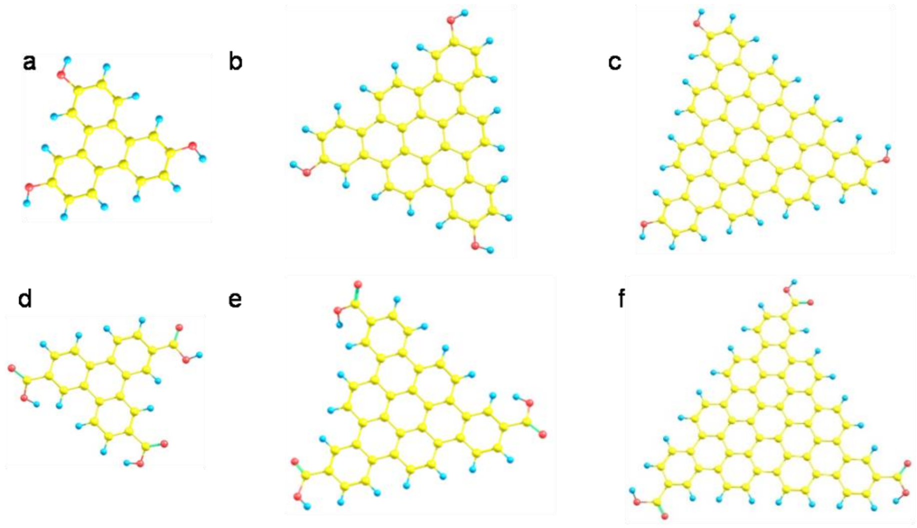

Supplementary Figure 45. The ball-and-stick model of CQDs with triangular structure functionalized with -OH (named T-CQDs-OH-1 (a), T-CQDs-OH-2 (b), and T-CQDs-OH-3 (c)) and -COOH groups (named T-CQDs-COOH-1 (d), T-CQDs-COOH-2 (e), and T-CQDs-COOH-3 (f)) at edge sites.

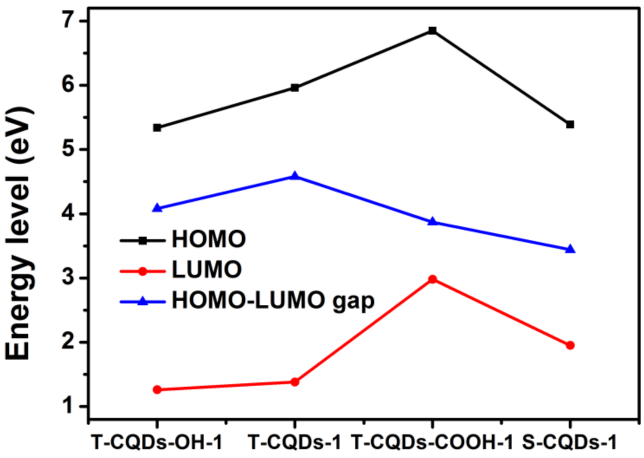

Supplementary Figure 46. The energy levels of T-CQDs-OH-1, T-CQDs-1, T-CQDs-COOH-1 and S-CQDs-1.

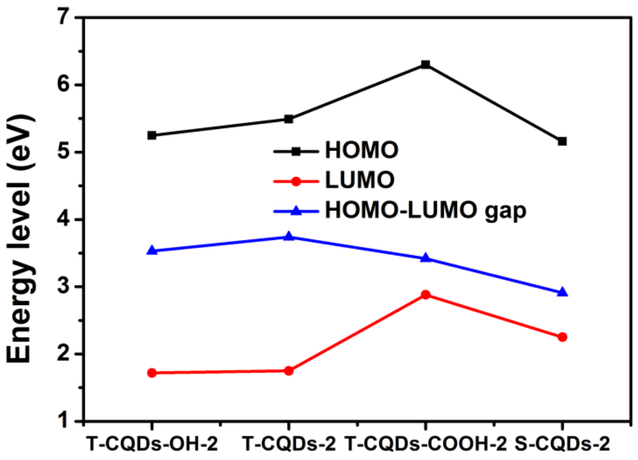

Supplementary Figure 47. The electron orbital energy levels of T-CQDs-OH-2, T-CQDs-2, T-CQDs-COOH-2 and S-CQDs-2.

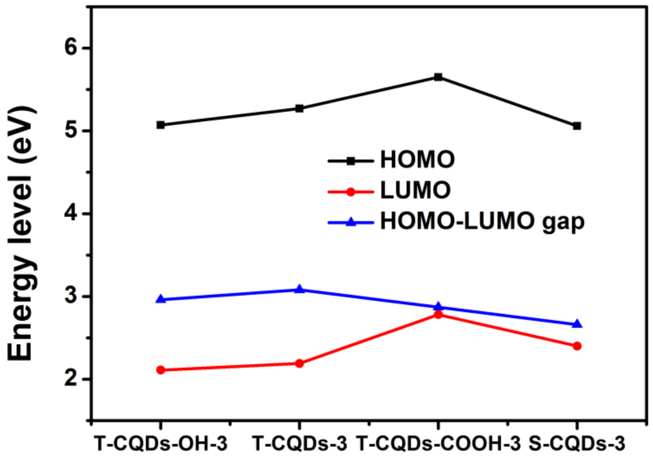

Supplementary Figure 48. The electron orbital energy levels of T-CQDs-OH-3, T-CQDs-3, T-CQDs-COOH-3 and S-CQDs-3.

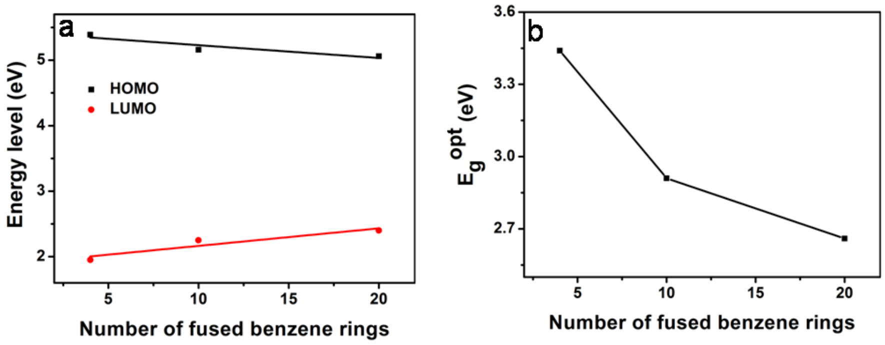

Supplementary Figure 49. The calculated HOMO and LUMO energy levels (a) and bandgap energies (b) of S-CQDs as a function of the number of fused benzene rings.

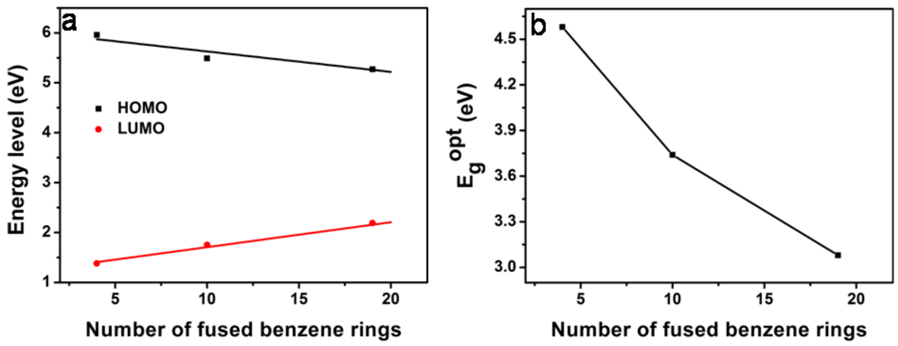

Supplementary Figure 50. The calculated HOMO and LUMO energy levels (a) and bandgap energies (b) of T-CQDs as a function of the number of fused benzene rings.

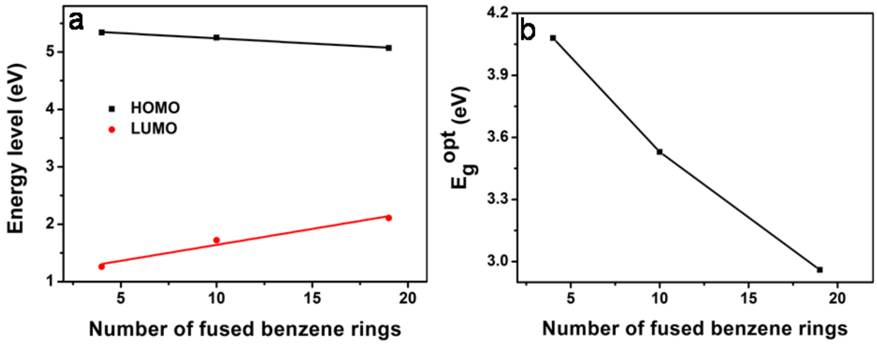

Supplementary Figure 51. The calculated HOMO and LUMO energy levels (a) and bandgap energies (b) of T-CQDs-OH as a function of the number of fused benzene rings.

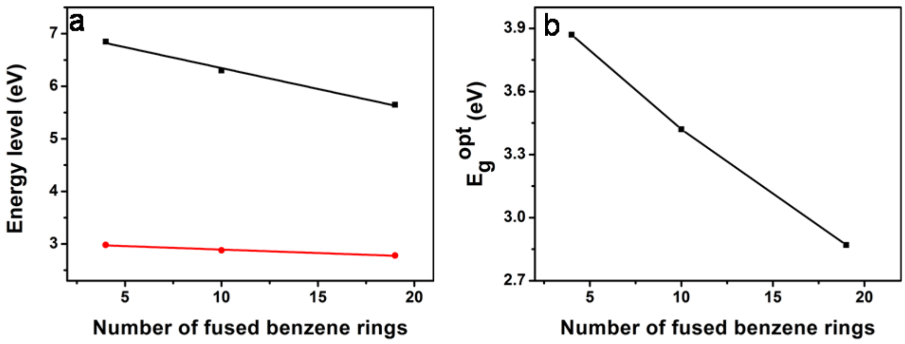

Supplementary Figure 52. The calculated HOMO and LUMO energy levels (a) and bandgap energies (b) of T-CQDs-COOH as a function of the number of fused benzene rings.

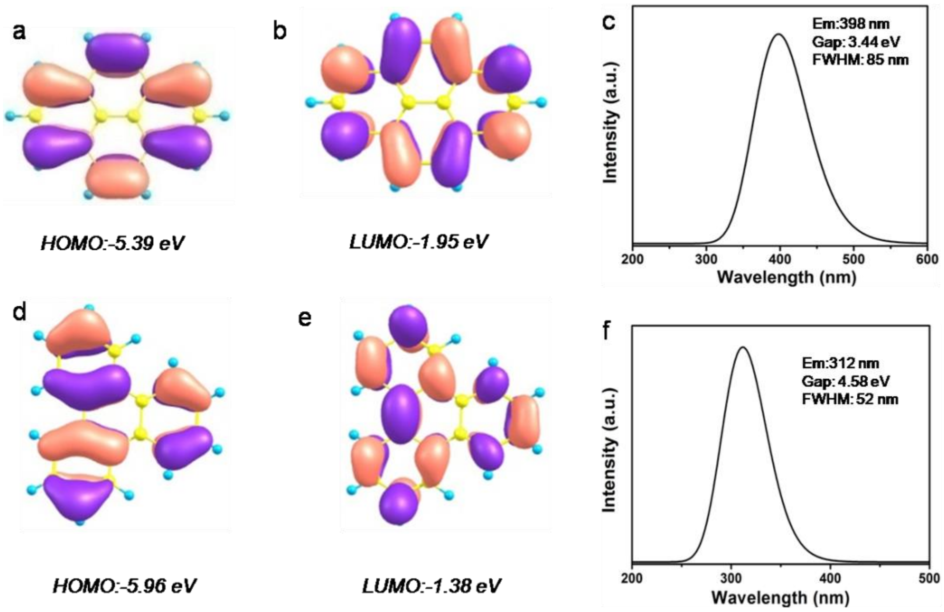

Supplementary Figure 53. The calculated molecular orbitals for HOMO (a) and LUMO (b) and PL spectra (c) of S-CQDs-1. The calculated molecular orbitals for HOMO (d) and LUMO (e) and PL spectra (f) of T-CQDs-1.

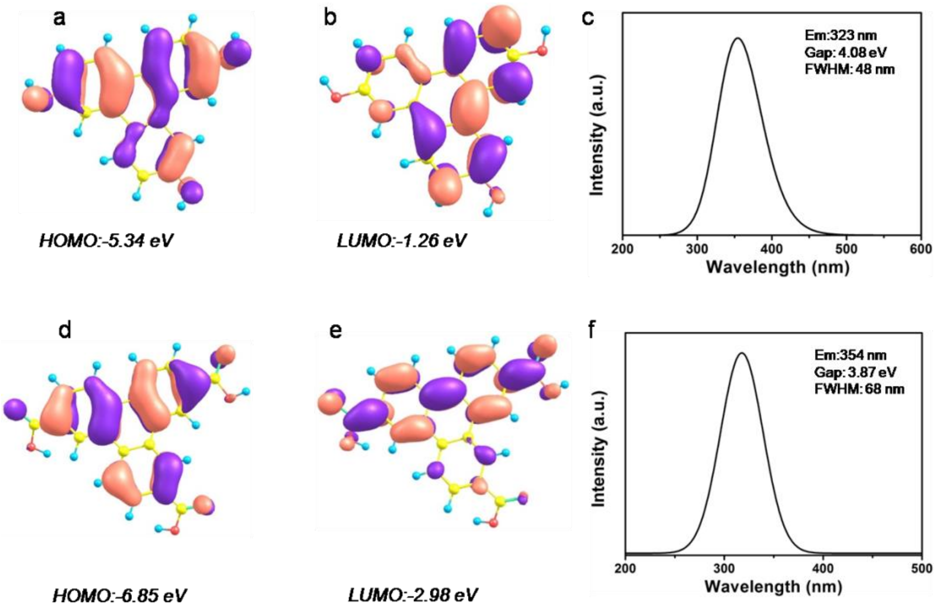

Supplementary Figure 54. The calculated molecular orbitals for HOMO (a) and LUMO (b) and PL spectra (c) of T-CQDs-OH-1. The calculated molecular orbitals for HOMO (d) and LUMO (e) and PL spectra (f) of T-CQDs-COOH-1.

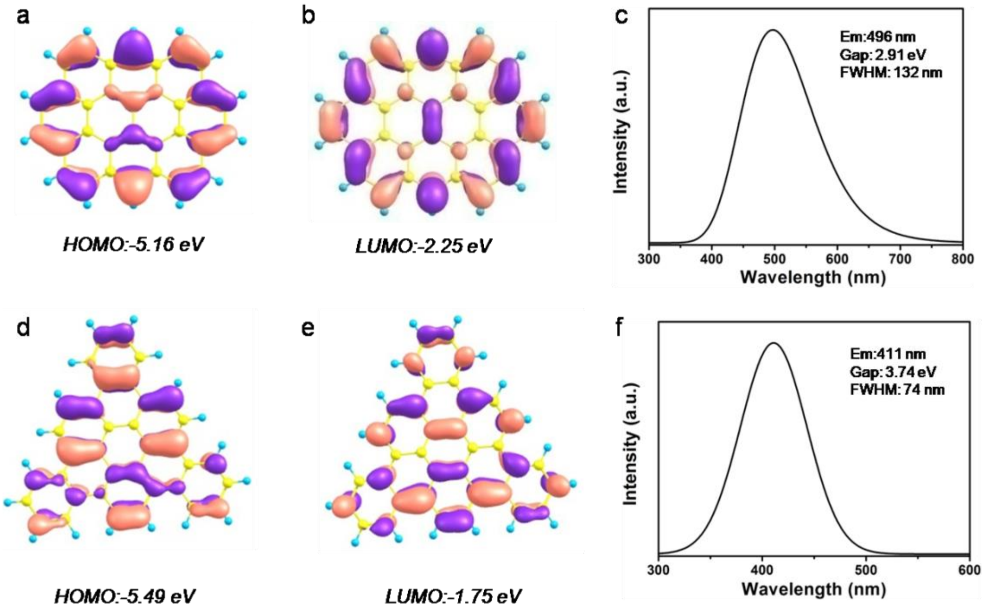

Supplementary Figure 55. The calculated molecular orbitals for HOMO (a) and LUMO (b) and PL spectra (c) of S-CQDs-2. The calculated molecular orbitals for HOMO (d) and LUMO (e) and PL spectra (f) of T-CQDs-2.

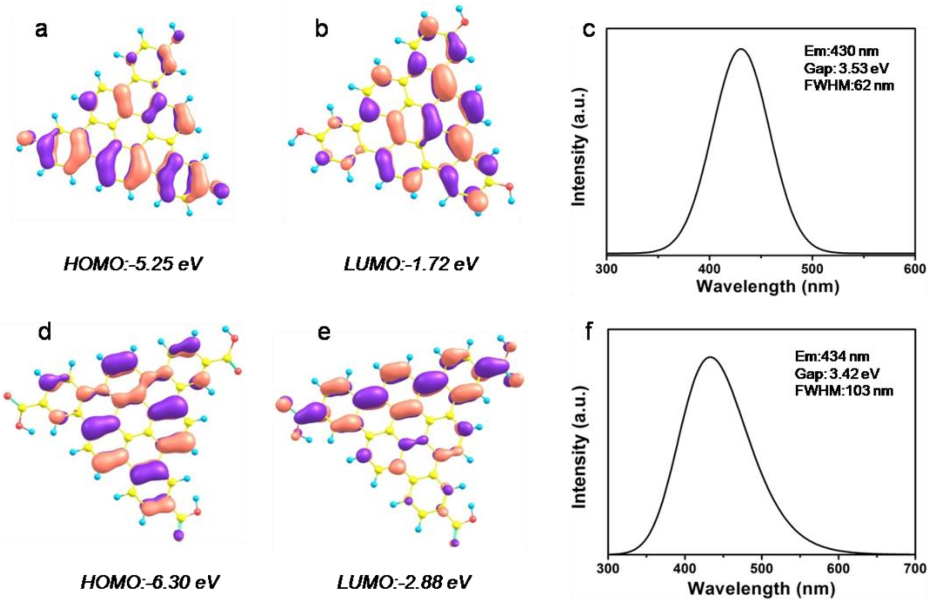

Supplementary Figure 56. The calculated molecular orbitals for HOMO (a) and LUMO (b) and PL spectra (c) of T-CQDs-OH-2. The calculated molecular orbitals for HOMO (d) and LUMO (e) and PL spectra (f) of T-CQDs-COOH-2.

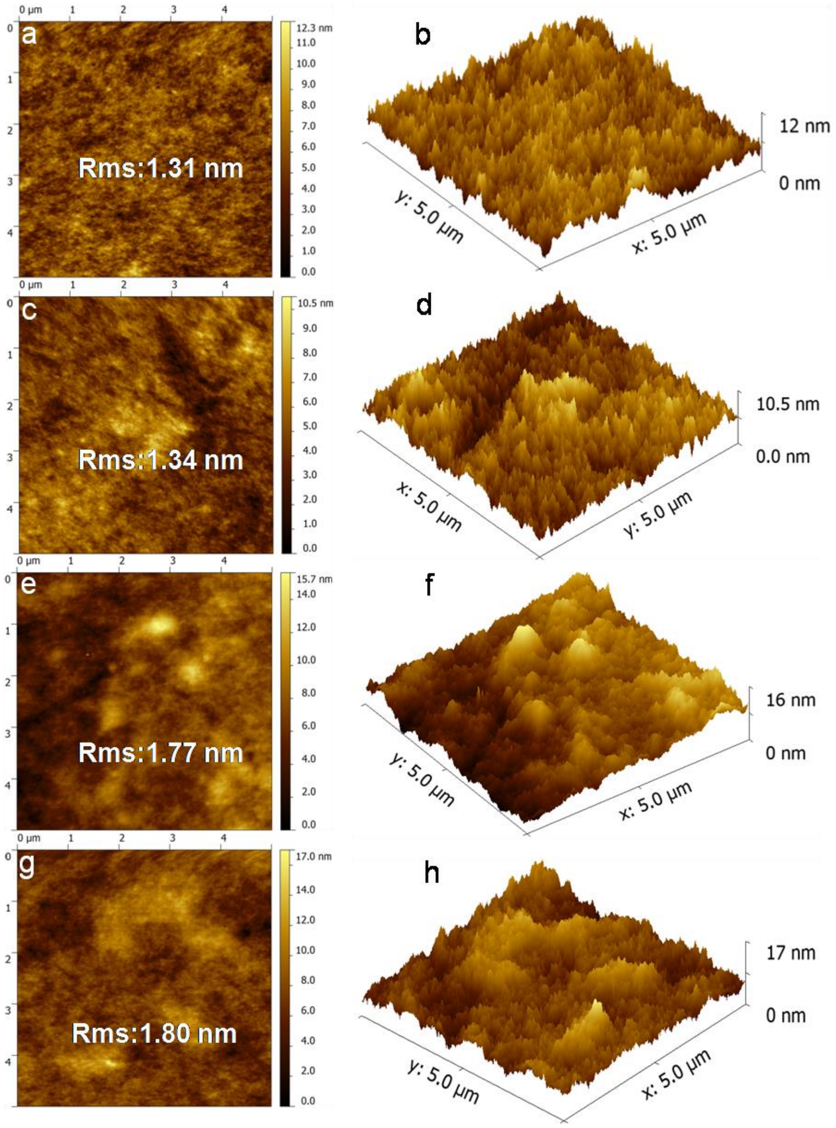

Supplementary Figure 57. The AFM height images of active emission layers of B- (a, b), G- (c, d), Y- (e, f), and R-LEDs (g, h).

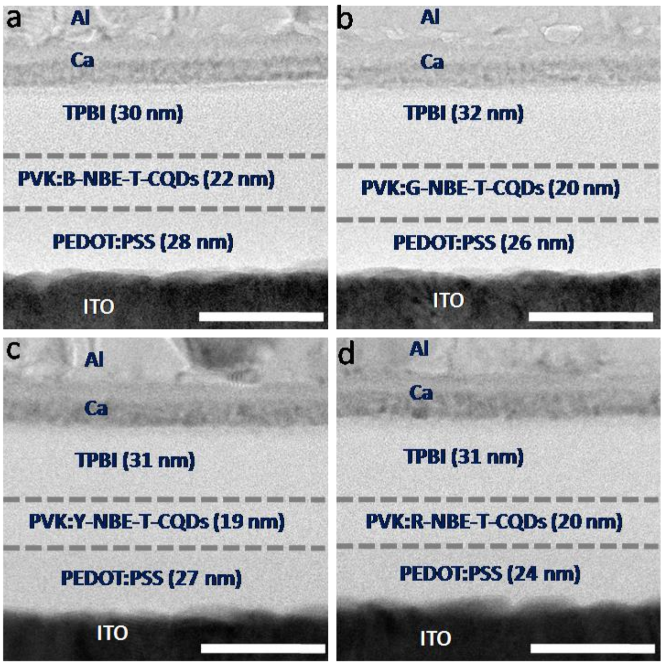

Supplementary Figure 58. The cross-sectional TEM images of B- (a), G- (b), Y- (c), and R-LED (d) devices based on the ITO/PEDOT:PSS/PVK:NBE-T-CQDs/TPBi /Ca/Al structure. Scale bar: 50 nm.

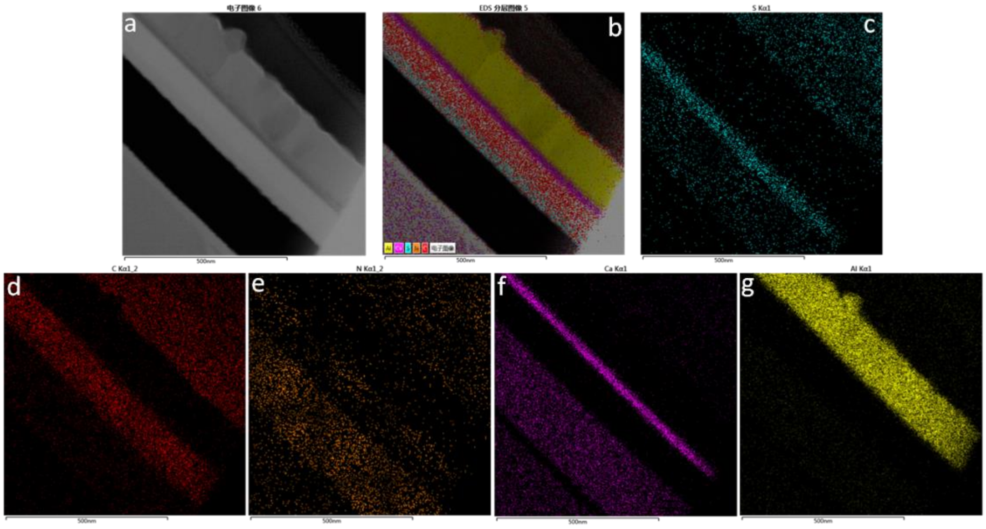

Supplementary Figure 59. The cross-sectional TEM image (a) and corresponding EDX map (b) of a typical LED device. The individual layers can be clearly distinguished based on their elemental composition such as S (c), C (d), N (e), Ca (f), and Al (g).

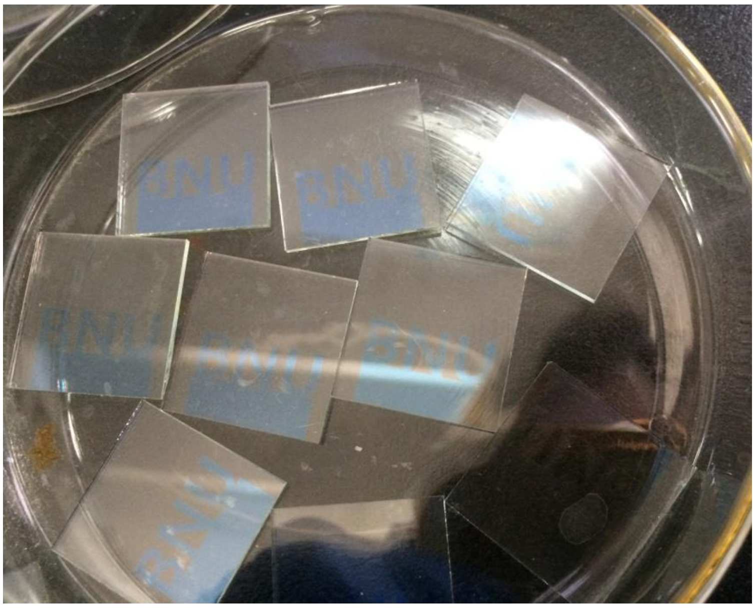

Supplementary Figure 60. The photographs of the ITO with Beijing Normal University(BNU) logo.

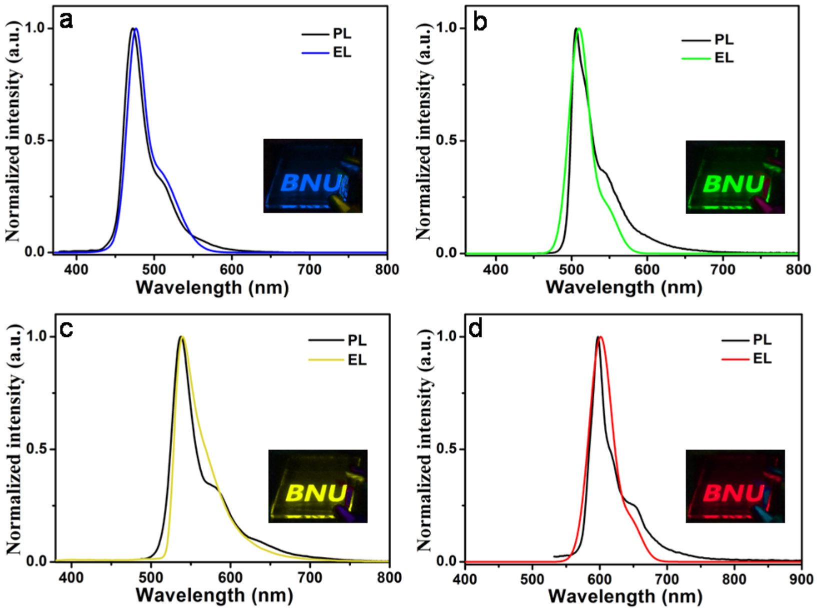

Supplementary Figure 61. The normalized PL spectra and the corresponding output EL spectra of B- (a), G- (b), Y- (c), and R-LEDs (d). The insets are the operation photographs of B-, G-, Y-, and R-LEDs with the logo of the BNU.

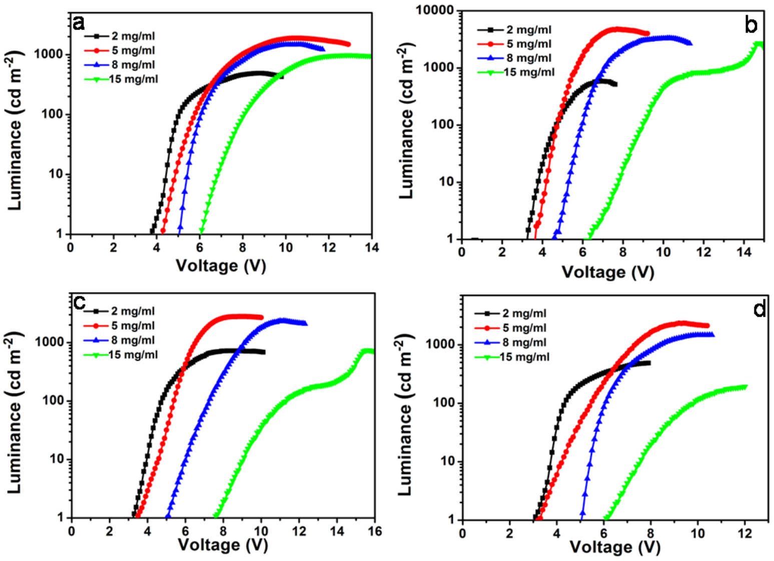

Supplementary Figure 62. The impact of theconcentration of NBE-T-CQDs blended PVK on the performance of B- (a), G- (b), Y- (c), and R-LEDs (d).

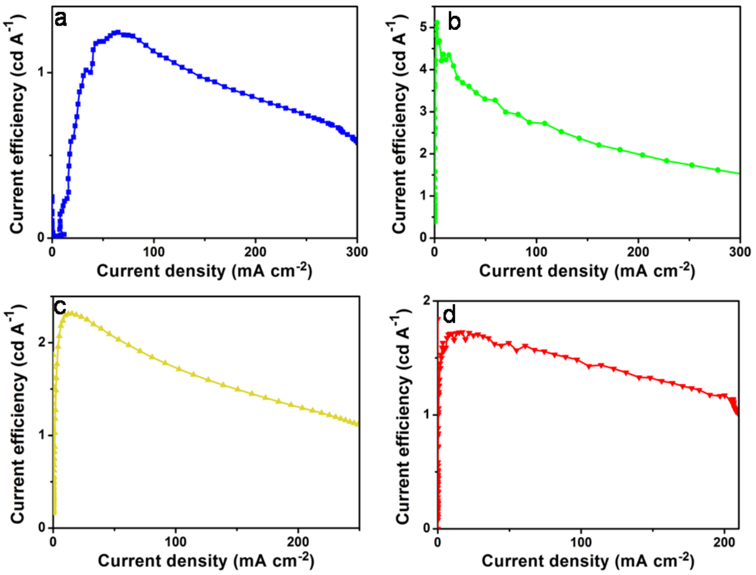

Supplementary Figure 63. The current efficiency vs current density of B- (a), G- (b), Y- (c), and R-LEDs (d).

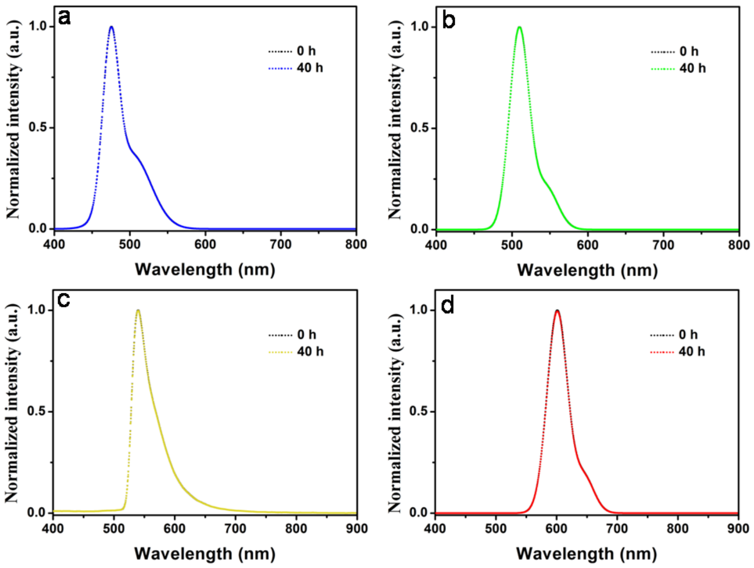

Supplementary Figure 64. The normalized EL spectra of B- (a), G- (b), Y- (c), and R-LEDs (d) at 0 and 40 h under operation at 6 V.

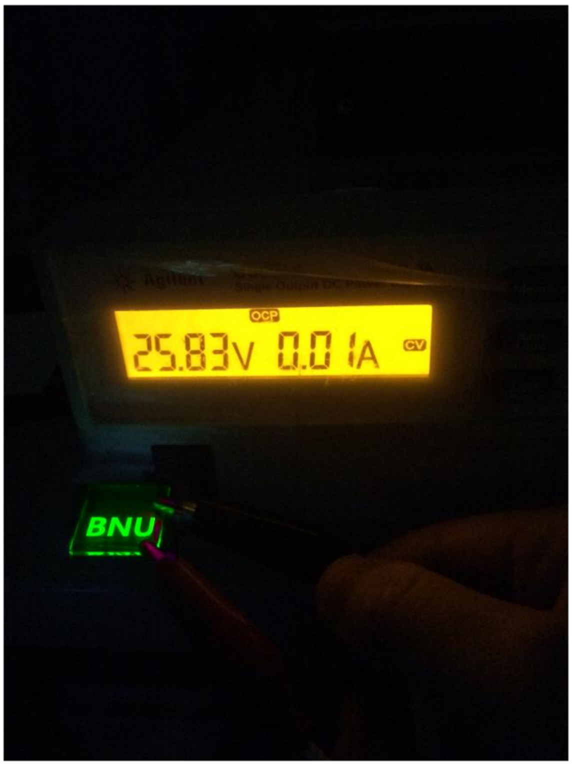

Supplementary Figure 65. The photograph of G-LEDs under operation at an extremely high voltage of 25.83 V.

Supplementary Tables

Supplementary Table 1. Estimation of the electron orbital energy levels of NBE-T-CQDs.

NBE-T-CQDs	HOMO (eV)	LUMO (eV)	E_g^{opt} (eV)
B-	5.18	2.55	2.63
G-	5.12	2.67	2.45
Y-	5.03	2.72	2.31
R-	4.92	2.85	2.07

Supplementary Table 2. Optimized Cartesian coordinates (Å) of S-CQDs-1 for the ground state (a) and the first excited state (b).

(a) ground state

C	0.679267000	-2.460340000	-0.000248000
C	-0.679218000	-2.460520000	-0.000097000
C	-1.427384000	-1.234600000	-0.000027000
C	-0.712540000	-0.000152000	-0.000155000
C	0.712668000	0.000161000	-0.000387000
C	1.427458000	-1.234477000	-0.000196000
H	-3.377050000	-2.146397000	0.000161000
H	1.226238000	-3.398801000	-0.000436000
H	-1.226000000	-3.399076000	0.000063000
C	-2.829336000	-1.208595000	0.000117000
C	-1.427595000	1.234372000	0.000052000
C	-2.829063000	1.208437000	0.000288000
C	-3.518686000	-0.000201000	0.000274000
H	-3.376928000	2.146189000	0.000506000
H	-4.604096000	0.000059000	0.000376000
C	0.679204000	2.460852000	-0.000430000
C	1.427240000	1.234727000	-0.000526000
C	-0.679100000	2.460851000	-0.000200000
H	1.226321000	3.399206000	-0.000330000
H	-1.227018000	3.398736000	0.000140000
C	2.829180000	1.208421000	0.000663000
C	3.518502000	-0.000210000	0.000170000
C	2.829538000	-1.208842000	0.000357000
H	3.376658000	2.146366000	0.000870000
H	4.603998000	0.001035000	0.000429000
H	3.377058000	-2.146625000	0.000287000

(b) first excited state

C	0.697348000	-2.447855000	0.000022000
C	-0.697226000	-2.447903000	0.000012000
C	-1.428221000	-1.240982000	0.000028000
C	-0.708021000	-0.000005000	0.000059000
C	0.707966000	0.000047000	0.000062000
C	1.428262000	-1.240868000	0.000049000
H	-3.401700000	-2.148658000	-0.000004000
H	1.236231000	-3.390353000	0.000010000
H	-1.236036000	-3.390444000	-0.000005000
C	-2.856560000	-1.209867000	0.000021000
C	-1.428317000	1.240907000	0.000080000
C	-2.856614000	1.209700000	0.000081000
C	-3.542916000	-0.000124000	0.000050000
H	-3.401860000	2.148429000	0.000115000
H	-4.628339000	-0.000135000	0.000057000
C	0.697162000	2.447949000	0.000119000
C	1.428169000	1.241019000	0.000095000
C	-0.697386000	2.447900000	0.000112000
H	1.235994000	3.390478000	0.000147000
H	-1.236279000	3.390394000	0.000137000
C	2.856496000	1.209919000	0.000106000

C	3.542863000	0.000171000	0.000072000
C	2.856569000	-1.209648000	0.000052000
H	3.401655000	2.148699000	0.000143000
H	4.628285000	0.000195000	0.000071000
H	3.401794000	-2.148390000	0.000029000

Supplementary Table 3. Optimized Cartesian coordinates (Å) of T-CQDs-1 for the ground state (a) and the first excited state (b).

(a) ground state

C	-1.922127000	3.234865000	0.000148000
C	-2.152601000	1.873965000	0.000089000
C	-1.096211000	0.937444000	0.000020000
C	0.237129000	1.422641000	-0.000196000
C	0.443693000	2.819126000	-0.000369000
C	-0.607825000	3.713205000	-0.000107000
H	-2.758705000	3.926118000	0.000290000
H	-3.179148000	1.531991000	-0.000163000
C	-1.350707000	-0.506128000	0.000160000
C	1.360396000	0.480632000	-0.000251000
H	1.449566000	3.217648000	-0.000483000
H	-0.411498000	4.780529000	-0.000307000
C	1.113734000	-0.916695000	-0.000340000
C	-0.263605000	-1.418043000	-0.000107000
C	-1.841300000	-3.281585000	-0.000465000
C	-0.547396000	-2.801011000	-0.000148000
C	-2.663431000	-1.025081000	0.000622000
C	-2.912701000	-2.382613000	-0.000082000
H	-2.021695000	-4.351703000	-0.000970000
H	0.261701000	-3.519446000	0.000861000
H	-3.510868000	-0.352564000	0.002651000
H	-3.935193000	-2.746107000	-0.000321000
C	2.219984000	-1.793745000	0.000023000
C	3.520139000	-1.330518000	0.000330000
C	3.763136000	0.046766000	0.000361000
C	2.699694000	0.926732000	-0.000005000
H	2.062258000	-2.864217000	-0.000430000
H	4.346090000	-2.034425000	0.000468000
H	4.779947000	0.425857000	0.000828000
H	2.917544000	1.986575000	-0.000531000

(b) first excited state

C	-1.930246000	3.242106000	0.001826000
C	-2.165881000	1.878855000	0.002932000
C	-1.115415000	0.923496000	0.000959000
C	0.258583000	1.424383000	-0.000717000
C	0.449533000	2.826901000	-0.002385000
C	-0.607780000	3.722969000	-0.001302000
H	-2.765068000	3.934753000	0.003420000
H	-3.194281000	1.542756000	0.005593000
C	-1.364199000	-0.486011000	0.000372000
C	1.359550000	0.500662000	-0.000411000
H	1.453505000	3.230530000	-0.004553000
H	-0.412145000	4.789830000	-0.002710000
C	1.106020000	-0.935757000	0.000547000
C	-0.243472000	-1.426994000	0.000099000
C	-1.843629000	-3.290494000	-0.001347000
C	-0.544195000	-2.810691000	-0.000736000
C	-2.677911000	-1.024548000	-0.000344000

C	-2.922500000	-2.385897000	-0.001162000
H	-2.024880000	-4.359906000	-0.002138000
H	0.260970000	-3.533674000	-0.001022000
H	-3.529810000	-0.357301000	-0.000524000
H	-3.943751000	-2.751577000	-0.001898000
C	2.226263000	-1.803522000	0.001693000
C	3.532096000	-1.335246000	0.001594000
C	3.775772000	0.046906000	0.000261000
C	2.708110000	0.933225000	-0.000664000
H	2.074122000	-2.874818000	0.002809000
H	4.357729000	-2.038746000	0.002633000
H	4.792075000	0.425972000	0.000148000
H	2.930835000	1.992052000	-0.001399000

Supplementary Table 4. Optimized Cartesian coordinates (Å) of T-CQDs-OH-1 for the ground state (a) and the first excited state (b).

(a) ground state

C	3.863411000	-0.417060000	-0.000012000
C	2.884875000	0.561995000	0.000255000
C	1.514625000	0.237051000	0.000192000
C	1.131952000	-1.130829000	-0.000103000
C	2.153380000	-2.103354000	-0.000474000
C	3.493143000	-1.769107000	-0.000424000
C	0.482877000	1.277746000	0.000267000
C	-0.285702000	-1.496141000	-0.000033000
C	-1.286396000	-0.488619000	-0.000056000
C	-0.893526000	0.925988000	-0.000063000
C	-1.501548000	3.286712000	-0.000164000
C	-1.859650000	1.949442000	-0.000321000
C	0.813714000	2.648790000	0.000498000
C	-0.145042000	3.641437000	0.000304000
C	-2.641865000	-0.868615000	0.000024000
C	-3.022625000	-2.199205000	0.000105000
C	-2.040957000	-3.200218000	0.000177000
C	-0.707456000	-2.842494000	0.000129000
O	5.168039000	-0.011394000	0.000103000
O	-2.503418000	4.212898000	-0.000492000
O	-4.359813000	-2.477712000	0.000159000
H	3.221778000	1.590237000	0.000469000
H	1.903314000	-3.156318000	-0.000866000
H	4.250407000	-2.547479000	-0.000731000
H	-2.917917000	1.725618000	-0.000674000
H	1.850646000	2.957747000	0.000841000
H	0.150226000	4.686501000	0.000485000
H	-3.434060000	-0.132030000	0.000073000
H	-2.324362000	-4.248888000	0.000308000
H	0.022251000	-3.641479000	0.000273000
H	5.754895000	-0.777498000	-0.000209000
H	-2.133498000	5.104260000	-0.000210000
H	-4.501391000	-3.432119000	0.000276000

(b) first excited state

C	3.864078000	-0.403769000	-0.000034000
C	2.882776000	0.584767000	0.000079000
C	1.503087000	0.260775000	0.000050000
C	1.106193000	-1.127991000	-0.000061000
C	2.137959000	-2.099092000	-0.000236000
C	3.489438000	-1.751801000	-0.000221000
C	0.496357000	1.275584000	0.000087000
C	-0.290159000	-1.503590000	0.000014000
C	-1.302889000	-0.487858000	-0.000013000
C	-0.907018000	0.904951000	0.000001000
C	-1.486020000	3.274344000	0.000033000
C	-1.859980000	1.939152000	-0.000038000
C	0.833572000	2.660335000	0.000174000
C	-0.117491000	3.646861000	0.000152000

C	-2.657421000	-0.867170000	-0.000029000
C	-3.026428000	-2.222654000	0.000000000
C	-2.058996000	-3.217647000	0.000078000
C	-0.700732000	-2.860505000	0.000091000
O	5.166827000	0.010486000	0.000023000
O	-2.471151000	4.203758000	-0.000014000
O	-4.368377000	-2.487500000	-0.000035000
H	3.226747000	1.609207000	0.000179000
H	1.890819000	-3.150762000	-0.000456000
H	4.247176000	-2.531562000	-0.000358000
H	-2.919769000	1.728437000	-0.000129000
H	1.872786000	2.958183000	0.000260000
H	0.167874000	4.695283000	0.000223000
H	-3.459814000	-0.143443000	-0.000046000
H	-2.345508000	-4.266595000	0.000131000
H	0.029576000	-3.657013000	0.000243000
H	5.751505000	-0.756051000	-0.000087000
H	-2.091525000	5.090924000	0.000041000
H	-4.503183000	-3.442182000	0.000005000

Supplementary Table 5. Optimized Cartesian coordinates (Å) of T-CQDs-COOH-1 for the ground state (a) and the first excited state (b).

(a) ground state

C	-4.029367000	-0.673483000	0.004272000
C	-3.061391000	0.319257000	0.018467000
C	-1.684527000	0.020478000	-0.003772000
C	-1.286018000	-1.341355000	-0.020283000
C	-2.288731000	-2.336290000	-0.047674000
C	-3.627693000	-2.018072000	-0.050271000
C	-0.674686000	1.081459000	-0.015640000
C	0.135277000	-1.689299000	-0.008853000
C	1.115222000	-0.663263000	0.000045000
C	0.704016000	0.744216000	-0.018673000
C	1.280607000	3.120107000	-0.045358000
C	1.649706000	1.783527000	-0.046719000
C	-1.031985000	2.445879000	-0.037845000
C	-0.085360000	3.448090000	-0.047850000
C	2.474511000	-1.027602000	0.027258000
C	2.872178000	-2.355653000	0.037714000
C	1.900635000	-3.368566000	0.022445000
C	0.565011000	-3.035184000	0.001410000
C	-5.495050000	-0.378254000	-0.015011000
O	-6.319025000	-1.155245000	-0.432437000
O	-5.898001000	0.823822000	0.457492000
C	2.373901000	4.139991000	-0.087028000
O	3.509202000	3.886370000	-0.411507000
O	2.056192000	5.408168000	0.258802000
C	4.306084000	-2.748969000	0.067394000
O	4.702004000	-3.894045000	0.071608000
O	5.144016000	-1.693744000	0.090287000
H	-3.370391000	1.355793000	0.008001000
H	-2.018844000	-3.382595000	-0.073952000
H	-4.379642000	-2.796525000	-0.084320000
H	2.708708000	1.570375000	-0.072071000
H	-2.071630000	2.741650000	-0.057465000
H	-0.426825000	4.476693000	-0.095919000
H	3.243709000	-0.270315000	0.043530000
H	2.211676000	-4.405886000	0.030859000
H	-0.157123000	-3.839346000	-0.003457000
H	-5.182852000	1.299283000	0.900808000
H	1.161513000	5.478255000	0.616927000
H	6.052555000	-2.032062000	0.108923000

(b) first excited state

C	-4.038655000	-0.667422000	0.043277000
C	-3.061900000	0.326821000	0.068582000
C	-1.691268000	0.040785000	-0.007928000
C	-1.264508000	-1.357561000	-0.074439000
C	-2.298638000	-2.349012000	-0.159341000
C	-3.622436000	-2.022708000	-0.120058000
C	-0.684697000	1.080614000	-0.031557000
C	0.108239000	-1.694004000	-0.049097000

C	1.126339000	-0.651888000	0.006969000
C	0.704958000	0.744180000	-0.022034000
C	1.274096000	3.148697000	-0.068674000
C	1.639809000	1.787082000	-0.061463000
C	-1.029735000	2.449444000	-0.090926000
C	-0.078241000	3.470051000	-0.104128000
C	2.472233000	-1.014908000	0.074388000
C	2.879724000	-2.352620000	0.071128000
C	1.893287000	-3.371791000	-0.002816000
C	0.565477000	-3.054840000	-0.059754000
C	-5.491815000	-0.389372000	0.085493000
O	-6.328294000	-1.166720000	-0.303487000
O	-5.875927000	0.826618000	0.583662000
C	2.376499000	4.156877000	-0.134532000
O	3.485809000	3.880349000	-0.505490000
O	2.070540000	5.425434000	0.234527000
C	4.291906000	-2.748942000	0.140826000
O	4.696023000	-3.894173000	0.133564000
O	5.142161000	-1.687388000	0.217360000
H	-3.380637000	1.361682000	0.103988000
H	-2.031546000	-3.391099000	-0.267811000
H	-4.388774000	-2.783897000	-0.201877000
H	2.700514000	1.581389000	-0.096647000
H	-2.070153000	2.740158000	-0.142907000
H	-0.420948000	4.496557000	-0.186830000
H	3.245426000	-0.262735000	0.133147000
H	2.221359000	-4.404409000	-0.007031000
H	-0.153454000	-3.861231000	-0.100382000
H	-5.150175000	1.233958000	1.072159000
H	1.207657000	5.468148000	0.665442000
H	6.037327000	-2.054461000	0.254835000

Supplementary Table 6. Optimized Cartesian coordinates (Å) of S-CQDs-2 for the ground state (a) and the first excited state (b).

(a) ground state

C	-3.692207000	-1.421291000	0.000000000
C	-2.461368000	-0.712370000	-0.000036000
C	-1.226437000	-1.424446000	-0.000142000
C	-1.222675000	-2.854418000	0.000029000
C	-2.482978000	-3.537247000	0.000091000
C	-3.660165000	-2.852529000	0.000090000
C	0.000002000	-0.716598000	0.000046000
C	-0.000016000	-3.534307000	0.000082000
C	1.222689000	-2.854427000	0.000028000
C	1.226442000	-1.424462000	-0.000157000
C	2.461362000	-0.712379000	-0.000001000
C	3.692193000	-1.421306000	-0.000005000
C	3.660166000	-2.852540000	0.000044000
C	2.482954000	-3.537263000	0.000057000
H	-2.482760000	-4.623263000	0.000134000
H	-4.603344000	-3.391215000	0.000174000
H	-0.000012000	-4.621037000	0.000154000
H	4.603323000	-3.391258000	0.000031000
H	2.482753000	-4.623278000	0.000093000
C	-4.900219000	0.688862000	-0.000140000
C	-3.692205000	1.421281000	-0.000145000
C	-2.461350000	0.712369000	-0.000161000
C	-4.900219000	-0.688864000	-0.000061000
C	-1.226437000	1.424452000	0.000035000
C	0.000003000	0.716593000	-0.000204000
C	1.226446000	1.424460000	-0.000008000
C	2.461371000	0.712370000	-0.000193000
H	-5.841895000	1.230007000	-0.000369000
H	-5.841900000	-1.230006000	-0.000031000
C	3.692197000	1.421306000	-0.000127000
C	4.900226000	0.688863000	0.000050000
C	4.900239000	-0.688853000	0.000099000
H	5.841890000	1.230038000	0.000133000
H	5.841906000	-1.230007000	0.000238000
C	-3.660178000	2.852533000	0.000071000
C	-2.482993000	3.537266000	0.000209000
C	-1.222680000	2.854425000	0.000178000
C	-0.000006000	3.534321000	0.000235000
C	1.222693000	2.854419000	0.000079000
C	2.482978000	3.537259000	-0.000064000
H	2.482769000	4.623273000	-0.000042000
C	3.660177000	2.852521000	-0.000143000
H	0.000011000	4.621046000	0.000368000
H	-4.603369000	3.391207000	0.000002000
H	-2.482770000	4.623278000	0.000460000
H	4.603358000	3.391209000	-0.000374000

(b) first excited state

C	-3.704078000	-1.426869000	0.000012000
C	-2.464892000	-0.712451000	0.000041000
C	-1.237157000	-1.420858000	-0.000014000
C	-1.231478000	-2.850824000	0.000027000
C	-2.481779000	-3.531446000	0.000026000
C	-3.673786000	-2.838253000	0.000016000
C	-0.000001000	-0.708164000	0.000081000
C	-0.000003000	-3.530757000	0.000032000
C	1.231474000	-2.850825000	0.000029000
C	1.237154000	-1.420859000	-0.000011000
C	2.464890000	-0.712453000	0.000048000
C	3.704076000	-1.426873000	0.000022000
C	3.673782000	-2.838256000	0.000021000
C	2.481774000	-3.531448000	0.000028000
H	-2.484911000	-4.617144000	0.000027000
H	-4.613943000	-3.382175000	0.000016000
H	-0.000003000	-4.617368000	0.000028000
H	4.613938000	-3.382179000	0.000023000
H	2.484905000	-4.617146000	0.000026000
C	-4.928217000	0.682009000	0.000007000
C	-3.704077000	1.426872000	0.000011000
C	-2.464892000	0.712453000	-0.000013000
C	-4.928218000	-0.682005000	0.000007000
C	-1.237156000	1.420859000	0.000048000
C	0.000000000	0.708164000	-0.000044000
C	1.237156000	1.420857000	0.000052000
C	2.464891000	0.712450000	-0.000005000
H	-5.866993000	1.227887000	0.000002000
H	-5.866994000	-1.227883000	0.000004000
C	3.704077000	1.426869000	0.000022000
C	4.928217000	0.682004000	0.000022000
C	4.928216000	-0.682009000	0.000020000
H	5.866993000	1.227883000	0.000019000
H	5.866992000	-1.227889000	0.000021000
C	-3.673783000	2.838256000	0.000011000
C	-2.481776000	3.531448000	0.000007000
C	-1.231476000	2.850825000	0.000008000
C	0.000001000	3.530757000	0.000007000
C	1.231477000	2.850824000	0.000012000
C	2.481777000	3.531445000	0.000015000
H	2.484910000	4.617143000	0.000018000
C	3.673785000	2.838252000	0.000022000
H	0.000002000	4.617367000	0.000014000
H	-4.613940000	3.382179000	0.000009000
H	-2.484907000	4.617146000	0.000010000
H	4.613941000	3.382175000	0.000024000

Supplementary Table 7. Optimized Cartesian coordinates (Å) of T-CQDs-2 for the ground state (a) and the first excited state (b).

(a) ground state

C	3.685019000	0.586610000	-0.000540000
C	3.224279000	1.878098000	-0.000595000
C	1.848347000	2.174520000	-0.000134000
C	0.932928000	1.092502000	-0.000018000
C	1.413799000	-0.255472000	0.000099000
C	2.807464000	-0.513760000	0.000067000
H	4.755340000	0.430824000	-0.001227000
H	3.954336000	2.676117000	-0.001033000
C	1.369892000	3.551003000	-0.000072000
C	-0.485964000	1.352024000	0.000140000
C	-0.958969000	2.688247000	0.000296000
C	-0.023918000	3.805964000	-0.000113000
C	0.423395000	6.204269000	-0.000620000
C	-0.464219000	5.149213000	-0.000578000
C	2.257653000	4.651065000	-0.000003000
C	1.801393000	5.952136000	-0.000213000
H	0.052817000	7.224191000	-0.000954000
H	-1.522583000	5.373442000	-0.001026000
H	3.326768000	4.485380000	0.000214000
H	2.509317000	6.774604000	-0.000118000
C	-2.350733000	2.898007000	0.001051000
C	-3.238811000	1.853168000	0.001210000
C	-2.807750000	0.513375000	0.000596000
C	-1.412856000	0.261708000	0.000279000
H	-2.751442000	3.902694000	0.001680000
H	-4.294870000	2.086759000	0.001886000
C	-0.928190000	-1.096848000	0.000283000
C	0.479647000	-1.354380000	0.000220000
C	5.162174000	-3.468179000	0.000174000
C	4.691853000	-2.172137000	0.000766000
C	3.308241000	-1.882315000	0.000275000
C	2.390648000	-2.961883000	-0.000200000
C	2.900014000	-4.280574000	-0.001116000
C	4.254978000	-4.535580000	-0.001011000
H	6.230763000	-3.657037000	0.000561000
H	5.414842000	-1.367326000	0.001773000
C	0.959237000	-2.688229000	0.000295000
H	2.222669000	-5.124165000	-0.002116000
H	4.613439000	-5.559836000	-0.001787000
C	-1.848831000	-2.174437000	0.000510000
C	-1.334450000	-3.484570000	0.001429000
C	0.014424000	-3.731426000	0.001291000
C	-3.284473000	-1.923732000	-0.000075000
H	-2.004081000	-4.333959000	0.002542000
H	0.339983000	-4.762961000	0.002217000
C	-3.760630000	-0.589143000	0.000191000
C	-5.157282000	-0.370552000	-0.000197000
C	-6.055807000	-1.416287000	-0.000873000
C	-5.585080000	-2.735682000	-0.001359000
C	-4.227549000	-2.976742000	-0.001020000
H	-5.548959000	0.637986000	-0.000119000

H	-7.122028000	-1.214412000	-0.001156000
H	-6.282957000	-3.566677000	-0.002100000
H	-3.892593000	-4.005530000	-0.001831000

(b) first excited state

C	3.404049000	1.553965000	-0.000494000
C	2.615139000	2.669252000	-0.000532000
C	1.192155000	2.588452000	-0.000198000
C	0.603014000	1.294311000	-0.000044000
C	1.423931000	0.128185000	0.000054000
C	2.855086000	0.249292000	-0.000125000
H	4.476396000	1.690665000	-0.000810000
H	3.101390000	3.634972000	-0.000862000
C	0.377791000	3.774808000	-0.000107000
C	-0.826012000	1.165166000	0.000226000
C	-1.638860000	2.333575000	0.000362000
C	-1.052341000	3.645251000	0.000129000
C	-1.257497000	6.083484000	-0.000073000
C	-1.837090000	4.827163000	0.000092000
C	0.935416000	5.078155000	-0.000224000
C	0.138043000	6.209529000	-0.000200000
H	-1.885835000	6.967931000	-0.000103000
H	-2.916417000	4.762756000	0.000139000
H	2.008633000	5.210272000	-0.000311000
H	0.597131000	7.192578000	-0.000294000
C	-3.056594000	2.157992000	0.000865000
C	-3.630176000	0.922309000	0.000877000
C	-2.852228000	-0.266133000	0.000417000
C	-1.423626000	-0.128404000	0.000245000
H	-3.707092000	3.021485000	0.001309000
H	-4.709402000	0.861862000	0.001330000
C	-0.600791000	-1.294462000	0.000352000
C	0.824593000	-1.165559000	0.000245000
C	5.925506000	-1.961559000	-0.000405000
C	5.115877000	-0.827875000	-0.000174000
C	3.707816000	-0.924383000	-0.000036000
C	3.109364000	-2.215446000	0.000061000
C	3.950659000	-3.344474000	-0.000369000
C	5.340042000	-3.221195000	-0.000583000
H	7.005101000	-1.854312000	-0.000500000
H	5.597124000	0.140618000	-0.000082000
C	1.655965000	-2.328616000	0.000592000
H	3.528891000	-4.340694000	-0.000689000
H	5.957060000	-4.114046000	-0.000898000
C	-1.208932000	-2.585716000	0.000591000
C	-0.357197000	-3.703751000	0.001594000
C	1.019283000	-3.579772000	0.001610000
C	-2.657044000	-2.734579000	-0.000090000
H	-0.770932000	-4.703260000	0.002561000
H	1.605174000	-4.488984000	0.002585000
C	-3.478800000	-1.569892000	-0.000042000
C	-4.883415000	-1.728164000	-0.000602000
C	-5.475357000	-2.987133000	-0.001242000
C	-4.671905000	-4.122700000	-0.001447000
C	-3.284177000	-3.996307000	-0.000924000

H	-5.530236000	-0.861569000	-0.000668000
H	-6.556520000	-3.076478000	-0.001665000
H	-5.119442000	-5.111436000	-0.002077000
H	-2.691154000	-4.900975000	-0.001388000

Supplementary Table 8. Optimized Cartesian coordinates (Å) of T-CQDs-OH-2 for the ground state (a) and the first excited state (b).

(a) ground state

C	3.145274000	2.040104000	-0.000154000
C	2.177144000	3.011635000	-0.000179000
C	0.806401000	2.691931000	-0.000031000
C	0.441643000	1.322030000	-0.000008000
C	1.451566000	0.308601000	0.000049000
C	2.821923000	0.670989000	0.000007000
H	4.180008000	2.355772000	-0.000374000
H	2.498118000	4.044944000	-0.000365000
C	-0.214874000	3.729071000	0.000035000
C	-0.953235000	0.952364000	-0.000007000
C	-1.952667000	1.957509000	-0.000087000
C	-1.586377000	3.368872000	-0.000064000
C	-2.212169000	5.720510000	0.000050000
C	-2.562257000	4.384468000	-0.000081000
C	0.109721000	5.102531000	0.000215000
C	-0.857225000	6.084655000	0.000250000
H	-3.619451000	4.158192000	-0.000239000
H	1.144519000	5.418387000	0.000399000
H	-0.569532000	7.133339000	0.000481000
C	-3.300817000	1.555480000	-0.000138000
C	-3.658476000	0.231578000	-0.000111000
C	-2.696226000	-0.794742000	-0.000040000
C	-1.326498000	-0.429481000	-0.000010000
H	-4.090422000	2.294609000	-0.000182000
H	-4.712920000	-0.009315000	-0.000097000
C	-0.307599000	-1.451095000	0.000135000
C	1.074721000	-1.084009000	0.000046000
C	6.210595000	-0.986239000	-0.000139000
C	5.228739000	-0.014945000	0.000135000
C	3.861046000	-0.351582000	0.000092000
C	3.487666000	-1.719709000	-0.000077000
C	4.514636000	-2.687646000	-0.000515000
C	5.848961000	-2.341730000	-0.000592000
H	5.561977000	1.013687000	0.000455000
C	2.078582000	-2.084730000	0.000239000
H	4.270110000	-3.741637000	-0.000928000
H	6.613393000	-3.115110000	-0.001041000
C	-0.681842000	-2.818261000	0.000318000
C	0.342666000	-3.783317000	0.000754000
C	1.668383000	-3.431078000	0.000699000
C	-2.086265000	-3.200434000	0.000188000
H	0.099808000	-4.837839000	0.001144000
H	2.402750000	-4.225790000	0.001157000
C	-3.089929000	-2.198804000	-0.000091000
C	-4.444551000	-2.584813000	-0.000263000
C	-4.814586000	-3.915399000	-0.000220000
C	-3.827250000	-4.912176000	0.000037000
C	-2.497167000	-4.550587000	0.000205000
H	-5.243834000	-1.856830000	-0.000559000
H	-4.107126000	-5.963049000	0.000061000
H	-1.761697000	-5.344056000	0.000335000

O	7.513712000	-0.575027000	-0.000087000
H	8.096904000	-1.342274000	0.000051000
O	-6.149555000	-4.206145000	-0.000414000
H	-6.274109000	-5.161767000	-0.000523000
O	-3.219184000	6.643350000	0.000108000
H	-2.846227000	7.531989000	-0.000517000

(b) first excited state

C	-3.145382000	2.050907000	0.000120000
C	-2.174958000	3.024645000	0.000136000
C	-0.799058000	2.709481000	0.000041000
C	-0.430220000	1.321047000	0.000002000
C	-1.443840000	0.309228000	-0.000023000
C	-2.829213000	0.678860000	0.000011000
H	-4.179388000	2.369751000	0.000220000
H	-2.496722000	4.057322000	0.000257000
C	0.215625000	3.734178000	-0.000003000
C	0.949978000	0.951868000	-0.000006000
C	1.962865000	1.971255000	0.000020000
C	1.600928000	3.364017000	0.000024000
C	2.219338000	5.723742000	0.000001000
C	2.573460000	4.389719000	0.000046000
C	-0.104730000	5.110692000	-0.000077000
C	0.860690000	6.093972000	-0.000083000
H	3.631575000	4.167169000	0.000103000
H	-1.139772000	5.426181000	-0.000163000
H	0.575333000	7.142944000	-0.000169000
C	3.313905000	1.554342000	0.000007000
C	3.667602000	0.227408000	-0.000009000
C	2.707091000	-0.806837000	-0.000018000
C	1.319821000	-0.429511000	-0.000042000
H	4.107556000	2.289299000	0.000000000
H	4.722626000	-0.013076000	-0.000034000
C	0.310186000	-1.436966000	-0.000126000
C	-1.072505000	-1.068041000	-0.000110000
C	-6.208109000	-0.982323000	0.000123000
C	-5.228900000	-0.006748000	0.000015000
C	-3.858568000	-0.334544000	0.000000000
C	-3.482930000	-1.718754000	-0.000002000
C	-4.515541000	-2.687691000	0.000190000
C	-5.848340000	-2.343196000	0.000260000
H	-5.566090000	1.020462000	-0.000060000
C	-2.089006000	-2.083034000	-0.000196000
H	-4.269854000	-3.741018000	0.000352000
H	-6.614060000	-3.114800000	0.000435000
C	0.683675000	-2.826738000	-0.000250000
C	-0.349895000	-3.789001000	-0.000546000
C	-1.676436000	-3.433134000	-0.000517000
C	2.073755000	-3.209702000	-0.000099000
H	-0.110708000	-4.843788000	-0.000835000
H	-2.411096000	-4.227152000	-0.000815000
C	3.089857000	-2.195370000	0.000009000
C	4.440941000	-2.600316000	0.000152000
C	4.798980000	-3.936643000	0.000193000
C	3.808957000	-4.936198000	0.000110000

C	2.481317000	-4.563279000	-0.000027000
H	5.248236000	-1.881131000	0.000258000
H	4.085272000	-5.987350000	0.000166000
H	1.740733000	-5.351902000	-0.000044000
O	-7.508460000	-0.575689000	0.000136000
H	-8.092038000	-1.343029000	0.000123000
O	6.128776000	-4.234029000	0.000329000
H	6.249319000	-5.190518000	0.000393000
O	3.222850000	6.649712000	0.000000000
H	2.845721000	7.536674000	0.000167000

Supplementary Table 9. Optimized Cartesian coordinates (Å) of T-CQDs-COOH-2 for the ground state (a) and the first excited state (b).

(a) ground state

C	-2.437755000	-4.633728000	-0.042213000
C	-3.772326000	-4.973928000	-0.040767000
C	-4.755315000	-3.967606000	-0.018899000
C	-4.355222000	-2.642961000	-0.023124000
C	-2.998459000	-2.268588000	-0.008113000
C	-2.012081000	-3.286888000	-0.015315000
C	-2.590449000	-0.869951000	0.007666000
C	-1.217041000	-0.523440000	0.005163000
C	-0.213658000	-1.559581000	-0.000415000
C	-0.602841000	-2.922933000	-0.005695000
C	-3.540155000	0.167584000	0.026304000
C	-3.163584000	1.486154000	0.031926000
C	-1.810197000	1.871121000	0.020008000
C	-0.825220000	0.852042000	0.013336000
C	1.173591000	-1.211378000	0.002493000
C	2.161063000	-2.228530000	0.005627000
C	1.735764000	-3.570442000	0.003058000
C	0.406252000	-3.904260000	-0.002864000
C	0.573151000	1.202883000	0.013963000
C	1.568548000	0.175439000	0.006383000
C	-1.418947000	3.274934000	0.019600000
C	-0.045177000	3.619376000	0.035733000
C	0.959403000	2.566514000	0.026325000
C	-2.373484000	4.314667000	0.001926000
C	-2.007822000	5.650206000	0.016732000
C	-0.643352000	5.983116000	0.073689000
C	0.306132000	4.989529000	0.068177000
C	2.943826000	0.518081000	0.003183000
C	3.963409000	-0.522998000	-0.007794000
C	3.575118000	-1.885072000	0.009634000
C	2.334229000	2.867741000	0.028116000
C	3.287634000	1.882616000	0.016497000
C	5.341125000	-0.216881000	-0.034298000
C	6.314756000	-1.201644000	-0.023742000
C	5.921414000	-2.549667000	0.037851000
C	4.586070000	-2.874683000	0.038440000
C	7.782686000	-0.897567000	-0.008135000
O	8.607455000	-1.655613000	0.421722000
O	8.158044000	0.309857000	-0.511865000
C	-6.229080000	-4.245781000	-0.041423000
O	-7.051054000	-3.436133000	-0.373811000
O	-6.616192000	-5.490755000	0.343106000
C	-3.004061000	6.770494000	0.041047000
O	-2.752875000	7.864506000	0.465264000
O	-4.243762000	6.493196000	-0.446660000
H	-1.711493000	-5.434206000	-0.076501000
H	-4.040001000	-6.024754000	-0.104481000
H	-5.142276000	-1.902495000	-0.042955000
H	-4.597484000	-0.058619000	0.039365000
H	-3.946898000	2.231739000	0.050709000
H	2.459497000	-4.373713000	0.001701000

H	0.148631000	-4.954619000	-0.003328000
H	-3.425677000	4.059712000	0.025070000
H	-0.361112000	7.027904000	0.119265000
H	1.346322000	5.283929000	0.099840000
H	2.669586000	3.895698000	0.038030000
H	4.325339000	2.187925000	0.023494000
H	5.647981000	0.821499000	-0.014220000
H	6.685067000	-3.316679000	0.080932000
H	4.320602000	-3.922468000	0.072778000
H	7.425961000	0.723436000	-0.986144000
H	-5.879115000	-5.978733000	0.730626000
H	-4.245546000	5.650041000	-0.916705000

(b) first excited state

C	2.418545000	-4.661634000	0.051803000
C	3.748937000	-4.995891000	0.051987000
C	4.745691000	-3.991954000	0.017590000
C	4.340148000	-2.660298000	0.016933000
C	2.992137000	-2.282803000	0.005464000
C	1.981898000	-3.304644000	0.016411000
C	2.581816000	-0.889138000	-0.012956000
C	1.203774000	-0.535894000	-0.009623000
C	0.201352000	-1.559050000	-0.003751000
C	0.603624000	-2.942456000	0.002644000
C	3.527501000	0.150055000	-0.036902000
C	3.160058000	1.482388000	-0.042752000
C	1.820404000	1.876584000	-0.025529000
C	0.818009000	0.845769000	-0.018499000
C	-1.172076000	-1.208708000	-0.006927000
C	-2.185819000	-2.227413000	-0.012285000
C	-1.749273000	-3.585798000	-0.012177000
C	-0.429935000	-3.923217000	-0.004008000
C	-0.562162000	1.197324000	-0.021487000
C	-1.561636000	0.171594000	-0.013646000
C	1.436082000	3.281988000	-0.020328000
C	0.059003000	3.631193000	-0.043274000
C	-0.946020000	2.585396000	-0.037837000
C	2.391341000	4.314915000	0.006885000
C	2.031134000	5.658678000	-0.007099000
C	0.671088000	5.994314000	-0.074601000
C	-0.285512000	5.001344000	-0.076691000
C	-2.941647000	0.520597000	-0.012403000
C	-3.964533000	-0.503484000	0.004520000
C	-3.568376000	-1.886196000	-0.017198000
C	-2.313918000	2.883533000	-0.046317000
C	-3.270736000	1.892146000	-0.034605000
C	-5.337325000	-0.194651000	0.040414000
C	-6.321666000	-1.174556000	0.033059000
C	-5.926671000	-2.532972000	-0.043909000
C	-4.601700000	-2.871144000	-0.052009000
C	-7.779373000	-0.872312000	0.028696000
O	-8.616935000	-1.648320000	-0.350568000
O	-8.152030000	0.362696000	0.478809000
C	6.209158000	-4.261893000	0.032916000
O	7.039734000	-3.421809000	0.271416000

O	6.603477000	-5.538117000	-0.242004000
C	3.029685000	6.775599000	-0.020131000
O	2.778788000	7.873424000	-0.435367000
O	4.269213000	6.492160000	0.463779000
H	1.693598000	-5.462857000	0.095933000
H	4.012371000	-6.047673000	0.123286000
H	5.132664000	-1.925006000	0.027415000
H	4.583996000	-0.079739000	-0.052938000
H	3.952494000	2.217891000	-0.066281000
H	-2.477672000	-4.384756000	-0.013378000
H	-0.175329000	-4.974244000	-0.006687000
H	3.442949000	4.056896000	-0.007681000
H	0.391497000	7.039685000	-0.121363000
H	-1.323839000	5.301451000	-0.114317000
H	-2.653345000	3.909874000	-0.061869000
H	-4.307049000	2.201359000	-0.048780000
H	-5.640884000	0.845119000	0.024101000
H	-6.697974000	-3.292174000	-0.090195000
H	-4.345820000	-3.920901000	-0.098130000
H	-7.421492000	0.785497000	0.946557000
H	5.865832000	-6.064221000	-0.573379000
H	4.271993000	5.649638000	0.934922000

Supplementary Table 10. Optimized Cartesian coordinates (Å) of S-CQDs-3 for the ground state (a) and the first excited state (b).

(a) ground state

C	7.128164000	-1.199720000	-0.238455000
C	5.733729000	-1.227624000	-0.110119000
C	5.016424000	0.000061000	-0.075857000
C	5.733548000	1.227536000	-0.119816000
C	7.128060000	1.198682000	-0.247418000
C	7.816786000	-0.000753000	-0.313914000
C	3.571947000	0.000158000	-0.016636000
C	2.860170000	1.228581000	0.002210000
C	3.582515000	2.477335000	0.005106000
C	5.005026000	2.489422000	-0.034733000
C	2.874747000	3.709464000	0.058480000
C	3.598037000	4.910595000	0.101551000
C	4.981314000	4.912821000	0.086431000
C	5.676889000	3.716216000	0.015376000
C	5.005413000	-2.488881000	-0.015199000
C	5.677449000	-3.714908000	0.049243000
C	4.981880000	-4.911054000	0.128080000
C	3.598516000	-4.909226000	0.135306000
C	2.875136000	-3.708757000	0.077713000
C	3.582809000	-2.476846000	0.019213000
C	1.421360000	-3.697933000	0.074807000
C	0.715096000	-2.471975000	0.052459000
C	1.436909000	-1.226757000	0.029717000
C	2.860307000	-1.228252000	0.007586000
C	0.722753000	0.000057000	0.032511000
C	1.436788000	1.226964000	0.026532000
C	0.714864000	2.472119000	0.049004000
C	1.420964000	3.698284000	0.065686000
C	0.685398000	-4.894555000	0.084615000
C	-0.684967000	-4.894695000	0.080868000
C	-1.421119000	-3.698218000	0.067292000
C	-0.714925000	-2.472129000	0.048233000
C	-1.436822000	-1.226934000	0.029122000
C	-0.722716000	-0.000067000	0.029513000
C	-1.436934000	1.226700000	0.032353000
C	-0.715157000	2.471964000	0.051798000
C	-1.421497000	3.697845000	0.076587000
C	-0.685627000	4.894514000	0.085793000
C	0.684732000	4.894718000	0.079957000
C	-2.860262000	1.228229000	0.006948000
C	-3.582847000	2.476734000	0.020721000
C	-2.875271000	3.708587000	0.080890000
C	-2.860133000	-1.228559000	0.001561000
C	-3.571922000	-0.000159000	-0.015800000
C	-2.874916000	-3.709321000	0.061572000
C	-3.582577000	-2.477229000	0.006619000
C	-3.598366000	-4.910248000	0.107887000
C	-4.981674000	-4.912277000	0.094014000
C	-5.677136000	-3.715765000	0.019915000
C	-5.005069000	-2.489240000	-0.033590000
C	-5.733330000	-1.227486000	-0.122398000

C	-5.016286000	-0.000048000	-0.077344000
C	-5.733496000	1.227618000	-0.112784000
C	-5.005428000	2.488712000	-0.014181000
C	-5.677644000	3.714452000	0.053525000
C	-4.982166000	4.910469000	0.135490000
C	-3.598780000	4.908835000	0.141657000
C	-7.127422000	-1.198667000	-0.254543000
C	-7.815852000	0.000810000	-0.323864000
C	-7.127510000	1.199796000	-0.245659000
H	7.692032000	-2.120648000	-0.297191000
H	7.692081000	2.119087000	-0.312235000
H	8.896227000	-0.001111000	-0.425145000
H	3.084713000	5.860538000	0.156309000
H	5.522625000	5.852301000	0.130724000
H	6.757726000	3.750407000	0.012519000
H	6.758320000	-3.748574000	0.052574000
H	5.523250000	-5.849846000	0.184531000
H	3.085117000	-5.858729000	0.196811000
H	1.191362000	-5.850048000	0.087023000
H	-1.190678000	-5.850320000	0.080595000
H	-1.191690000	5.849961000	0.088199000
H	1.190362000	5.850381000	0.079723000
H	-3.085136000	-5.860104000	0.165103000
H	-5.523088000	-5.851550000	0.141292000
H	-6.758004000	-3.749617000	0.018515000
H	-6.758536000	3.747769000	0.058185000
H	-5.523618000	5.849035000	0.194827000
H	-3.085441000	5.858211000	0.205722000
H	-7.691059000	-2.119153000	-0.321797000
H	-8.894864000	0.001184000	-0.439177000
H	-7.690967000	2.120831000	-0.306881000

(b) first excited state

C	7.142725000	1.199414000	0.025058000
C	5.740271000	1.230728000	0.026616000
C	5.018351000	-0.000012000	0.000378000
C	5.740261000	-1.230768000	-0.025394000
C	7.142711000	-1.199510000	-0.022096000
C	7.834620000	-0.000062000	0.002014000
C	3.582004000	0.000001000	-0.000112000
C	2.867216000	-1.234634000	-0.014867000
C	3.583648000	-2.474135000	-0.039138000
C	5.011192000	-2.488477000	-0.053146000
C	2.872247000	-3.713044000	-0.053556000
C	3.597779000	-4.915474000	-0.094266000
C	4.981985000	-4.917651000	-0.115344000
C	5.680218000	-3.720775000	-0.092791000
C	5.011210000	2.488473000	0.052977000
C	5.680249000	3.720800000	0.091470000
C	4.982033000	4.917706000	0.112886000
C	3.597823000	4.915518000	0.092169000
C	2.872272000	3.713063000	0.052567000
C	3.583665000	2.474141000	0.038742000
C	1.426646000	3.702202000	0.024905000
C	0.718572000	2.466288000	0.009497000

C	1.433912000	1.234691000	0.007886000
C	2.867221000	1.234632000	0.015304000
C	0.713791000	0.000004000	0.000671000
C	1.433902000	-1.234673000	-0.008958000
C	0.718575000	-2.466278000	-0.009118000
C	1.426632000	-3.702190000	-0.025452000
C	0.685989000	4.898025000	0.013048000
C	-0.685985000	4.898028000	-0.012078000
C	-1.426645000	3.702208000	-0.024119000
C	-0.718571000	2.466290000	-0.008966000
C	-1.433912000	1.234693000	-0.007613000
C	-0.713791000	0.000005000	-0.000657000
C	-1.433902000	-1.234674000	0.008714000
C	-0.718575000	-2.466280000	0.008636000
C	-1.426631000	-3.702195000	0.024736000
C	-0.685980000	-4.898018000	0.012488000
C	0.685982000	-4.898015000	-0.013375000
C	-2.867216000	-1.234637000	0.014605000
C	-3.583649000	-2.474143000	0.038600000
C	-2.872248000	-3.713055000	0.052782000
C	-2.867220000	1.234635000	-0.015036000
C	-3.582004000	0.000002000	0.000105000
C	-2.872271000	3.713076000	-0.051726000
C	-3.583665000	2.474150000	-0.038189000
C	-3.597824000	4.915541000	-0.090988000
C	-4.982035000	4.917735000	-0.111667000
C	-5.680249000	3.720823000	-0.090563000
C	-5.011210000	2.488486000	-0.052412000
C	-5.740270000	1.230734000	-0.026388000
C	-5.018351000	-0.000012000	-0.000415000
C	-5.740261000	-1.230774000	0.025053000
C	-5.011193000	-2.488488000	0.052558000
C	-5.680220000	-3.720794000	0.091919000
C	-4.981988000	-4.917676000	0.114231000
C	-3.597781000	-4.915494000	0.093200000
C	-7.142725000	1.199418000	-0.024905000
C	-7.834620000	-0.000064000	-0.002171000
C	-7.142711000	-1.199516000	0.021702000
H	7.711957000	2.118485000	0.041067000
H	7.711921000	-2.118613000	-0.037085000
H	8.919723000	-0.000083000	0.002787000
H	3.084111000	-5.866489000	-0.114411000
H	5.521544000	-5.858513000	-0.149308000
H	6.760550000	-3.758046000	-0.109612000
H	6.760587000	3.758092000	0.107767000
H	5.521607000	5.858595000	0.145832000
H	3.084176000	5.866564000	0.111375000
H	1.190101000	5.854378000	0.019677000
H	-1.190094000	5.854383000	-0.018572000
H	-1.190100000	-5.854369000	0.018911000
H	1.190104000	-5.854364000	-0.019929000
H	-3.084178000	5.866593000	-0.109932000
H	-5.521609000	5.858633000	-0.144335000
H	-6.760588000	3.758120000	-0.106818000
H	-6.760552000	-3.758069000	0.108702000
H	-5.521548000	-5.858545000	0.147966000
H	-3.084114000	-5.866514000	0.113138000

H	-7.711958000	2.118492000	-0.040740000
H	-8.919723000	-0.000085000	-0.002999000
H	-7.711921000	-2.118623000	0.036453000

Supplementary Table 11. Optimized Cartesian coordinates (Å) of T-CQDs-3 for the ground state (a) and the first excited state (b).

(a) ground state

C	-6.506231000	-5.752729000	0.002360000
C	-6.168769000	-4.416359000	0.002359000
C	-4.820764000	-3.989101000	0.000686000
C	-3.799085000	-4.971812000	-0.001057000
C	-4.173915000	-6.335386000	-0.000806000
C	-5.496206000	-6.724281000	0.000822000
C	-4.459352000	-2.579164000	0.000877000
C	-3.098818000	-2.184363000	0.000167000
C	-2.062434000	-3.181198000	-0.001108000
C	-2.404281000	-4.556090000	-0.002606000
C	-5.442205000	-1.569802000	0.001927000
C	-5.108882000	-0.241679000	0.001868000
C	-3.765424000	0.185575000	0.000806000
C	-2.747888000	-0.797233000	0.000295000
C	-0.690034000	-2.776797000	-0.000895000
C	0.331615000	-3.755325000	-0.001827000
C	-0.043288000	-5.114368000	-0.005415000
C	-1.357457000	-5.498963000	-0.005798000
C	-3.418013000	1.590528000	0.000071000
C	-2.059784000	1.985910000	0.000143000
C	-1.024690000	0.985312000	0.000000000
C	-1.365839000	-0.394282000	0.000249000
C	-0.340908000	-1.380085000	-0.000321000
C	1.024341000	-0.985743000	0.000102000
C	2.064465000	-1.981131000	0.000211000
C	1.721964000	-3.353743000	0.000564000
C	-4.407607000	2.594638000	-0.000782000
C	-4.083564000	3.925048000	-0.001116000
C	-2.743616000	4.360092000	-0.000614000
C	-1.723772000	3.376728000	-0.000261000
C	3.441123000	-1.591481000	0.000847000
C	4.463451000	-2.572264000	0.001681000
C	4.080630000	-3.928111000	0.004379000
C	2.763813000	-4.303536000	0.003860000
C	0.341464000	1.379917000	0.000437000
C	1.365720000	0.394769000	0.000017000
C	-2.406249000	5.775965000	-0.000688000
C	-1.044387000	6.169324000	-0.000750000
C	-0.003971000	5.151488000	-0.000396000
C	-0.342338000	3.775742000	-0.000053000
C	3.786320000	-0.195535000	0.000865000
C	5.147801000	0.195888000	0.001720000
C	6.205401000	-0.804093000	0.000715000
C	5.865108000	-2.180275000	0.000227000
C	-3.399850000	6.782228000	-0.000733000
C	-3.075577000	8.121849000	-0.001037000
C	-1.729177000	8.510762000	-0.001278000
C	-0.740481000	7.550444000	-0.001084000
C	7.573610000	-0.446798000	-0.000443000
C	8.571666000	-1.397388000	-0.002259000
C	8.235286000	-2.757878000	-0.003337000
C	6.909295000	-3.133944000	-0.002174000

C	1.361577000	5.497899000	-0.000337000
C	2.345129000	4.545194000	0.000224000
C	2.043411000	3.168051000	0.000685000
C	0.683548000	2.778355000	0.000195000
C	2.749766000	0.790787000	0.000838000
C	3.086490000	2.164813000	0.001349000
C	4.450842000	2.519717000	0.002913000
C	5.441013000	1.573901000	0.003147000
H	-7.550429000	-6.047971000	0.003693000
H	-6.968930000	-3.688164000	0.003818000
H	-3.415391000	-7.106876000	-0.001508000
H	-5.750692000	-7.779167000	0.001047000
H	-6.492124000	-1.829613000	0.002721000
H	-5.912602000	0.481737000	0.002840000
H	0.710688000	-5.889508000	-0.008670000
H	-1.576114000	-6.558249000	-0.008971000
H	-5.455815000	2.329076000	-0.001405000
H	-4.891476000	4.644174000	-0.001949000
H	4.830459000	-4.707617000	0.007585000
H	2.539342000	-5.361345000	0.006894000
H	-4.447135000	6.510769000	-0.000422000
H	-3.861865000	8.869708000	-0.001088000
H	-1.462829000	9.562715000	-0.001559000
H	0.290204000	7.879444000	-0.001219000
H	7.862290000	0.595905000	-0.000237000
H	9.612439000	-1.090285000	-0.003181000
H	9.013091000	-3.514551000	-0.005307000
H	6.678994000	-4.191080000	-0.003788000
H	1.661674000	6.537046000	-0.000660000
H	3.373479000	4.879606000	0.000073000
H	4.745077000	3.560277000	0.004230000
H	6.467735000	1.914066000	0.004463000

(b) first excited state

C	7.354304000	-4.634920000	0.001147000
C	6.803151000	-3.367985000	0.001798000
C	5.406294000	-3.162569000	0.000892000
C	4.548843000	-4.299427000	-0.000826000
C	5.142624000	-5.584914000	-0.001504000
C	6.511926000	-5.754211000	-0.000687000
C	4.822622000	-1.830632000	0.001268000
C	3.407934000	-1.658655000	-0.000722000
C	2.548558000	-2.803360000	0.000536000
C	3.113608000	-4.113161000	-0.001209000
C	5.628510000	-0.675727000	0.002432000
C	5.087568000	0.585508000	0.001647000
C	3.692675000	0.793910000	0.000057000
C	2.840387000	-0.346031000	0.002213000
C	1.131594000	-2.624436000	-0.002239000
C	0.269744000	-3.759799000	-0.000754000
C	0.866173000	-5.043369000	-0.002358000
C	2.224900000	-5.210371000	-0.003162000
C	3.124857000	2.117138000	0.000205000
C	1.710785000	2.290455000	-0.001964000
C	0.854927000	1.144666000	0.003754000
C	1.417700000	-0.170855000	-0.003748000
C	0.565444000	-1.308689000	0.003590000

C	-0.858171000	-1.137756000	-0.003812000
C	-1.721680000	-2.282190000	0.001850000
C	-1.154764000	-3.589450000	-0.000171000
C	3.937585000	3.270937000	-0.001374000
C	3.404772000	4.532591000	-0.002275000
C	2.008339000	4.748871000	-0.001207000
C	1.156721000	3.609178000	0.000619000
C	-3.140438000	-2.118469000	-0.000767000
C	-4.000520000	-3.258031000	0.000779000
C	-3.398489000	-4.532675000	0.002124000
C	-2.037695000	-4.694850000	0.001085000
C	-0.557093000	1.314789000	-0.003474000
C	-1.416508000	0.169867000	0.003938000
C	1.449805000	6.084727000	-0.001146000
C	0.034498000	6.253654000	0.000327000
C	-0.823200000	5.090303000	0.000816000
C	-0.266992000	3.780214000	-0.000651000
C	-3.702943000	-0.803672000	0.001155000
C	-5.119826000	-0.635714000	-0.000321000
C	-6.003868000	-1.791147000	-0.000216000
C	-5.442210000	-3.099004000	0.000678000
C	2.267321000	7.240115000	-0.002031000
C	1.731773000	8.509479000	-0.001685000
C	0.337099000	8.676510000	-0.000148000
C	-0.485129000	7.571925000	0.000688000
C	-7.409209000	-1.659695000	-0.000642000
C	-8.245863000	-2.760955000	-0.000359000
C	-7.694663000	-4.046722000	0.000554000
C	-6.322056000	-4.206040000	0.000922000
C	-2.232688000	5.209947000	0.002231000
C	-3.048348000	4.111622000	0.001711000
C	-2.532772000	2.795476000	0.000422000
C	-1.116910000	2.630661000	0.002165000
C	-2.841265000	0.336681000	-0.001736000
C	-3.397507000	1.647115000	0.000708000
C	-4.803552000	1.775101000	-0.000084000
C	-5.626991000	0.677256000	-0.000884000
H	8.432221000	-4.757979000	0.001885000
H	7.476304000	-2.520713000	0.003192000
H	4.519533000	-6.469395000	-0.003045000
H	6.931983000	-6.754909000	-0.001260000
H	6.706412000	-0.763739000	0.003150000
H	5.766802000	1.426837000	0.002899000
H	0.247794000	-5.930427000	-0.004520000
H	2.609729000	-6.221504000	-0.003877000
H	5.014888000	3.177791000	-0.002482000
H	4.086825000	5.371692000	-0.002908000
H	-4.012710000	-5.423412000	0.002665000
H	-1.648697000	-5.703757000	0.002881000
H	3.344701000	7.141655000	-0.003233000
H	2.386588000	9.374548000	-0.002400000
H	-0.094029000	9.672273000	0.000156000
H	-1.554987000	7.732284000	0.001919000
H	-7.862218000	-0.676985000	-0.001411000
H	-9.322207000	-2.624019000	-0.000719000
H	-8.339424000	-4.919717000	0.000784000
H	-5.926339000	-5.213130000	0.001698000

H	-2.696580000	6.186796000	0.002604000
H	-4.117119000	4.275989000	0.003076000
H	-5.264364000	2.753180000	-0.000974000
H	-6.695256000	0.847364000	-0.001022000

Supplementary Table 12. Optimized Cartesian coordinates (Å) of T-CQDs-OH-3 for the ground state (a) and the first excited state (b).

(a) ground state

C	1.547343000	8.499908000	-0.000755000
C	0.582363000	7.521493000	-0.000538000
C	0.902374000	6.142611000	-0.000254000
C	2.270270000	5.776073000	-0.000248000
C	3.249923000	6.794502000	-0.000535000
C	2.901991000	8.130727000	-0.000757000
C	-0.120954000	5.111546000	-0.000004000
C	0.238247000	3.740769000	0.000058000
C	1.625925000	3.366290000	0.000121000
C	2.630161000	4.366296000	0.000040000
C	-1.492595000	5.435282000	0.000112000
C	-2.460038000	4.466231000	0.000319000
C	-2.136838000	3.093821000	0.000448000
C	-0.770698000	2.726941000	0.000335000
C	1.985620000	1.981047000	0.000144000
C	3.350454000	1.608515000	0.000352000
C	4.323680000	2.628742000	0.000418000
C	3.976789000	3.953181000	0.000252000
C	-3.163260000	2.073841000	0.000744000
C	-2.803988000	0.705186000	0.000355000
C	-1.413266000	0.332310000	0.000344000
C	-0.405496000	1.334465000	0.000102000
C	0.967276000	0.963068000	0.000327000
C	1.331514000	-0.410715000	0.000032000
C	2.720388000	-0.790187000	0.000268000
C	3.721500000	0.209623000	0.000377000
C	-4.533309000	2.406383000	0.001345000
C	-5.507487000	1.443984000	0.001236000
C	-5.191087000	0.071769000	0.000575000
C	-3.823771000	-0.298515000	0.000360000
C	3.094383000	-2.171436000	0.000094000
C	4.461951000	-2.541058000	0.000244000
C	5.427887000	-1.516262000	0.000563000
C	5.072313000	-0.193929000	0.000606000
C	-1.048708000	-1.042386000	0.000014000
C	0.323166000	-1.413625000	0.000216000
C	-6.234122000	-0.944381000	0.000108000
C	-5.866825000	-2.314721000	-0.000135000
C	-4.461223000	-2.685340000	0.000125000
C	-3.454465000	-1.688565000	0.000135000
C	2.074102000	-3.185137000	-0.000029000
C	2.440342000	-4.553743000	-0.000218000
C	3.841072000	-4.942304000	-0.000325000
C	4.849683000	-3.944727000	0.000022000
C	-7.600587000	-0.601233000	-0.000222000
C	-8.586304000	-1.568121000	-0.000805000
C	-8.230521000	-2.925965000	-0.001048000
C	-6.898633000	-3.278500000	-0.000740000
C	4.245771000	-6.294990000	-0.000719000
C	5.573696000	-6.662084000	-0.000693000

C	6.566134000	-5.669332000	-0.000251000
C	6.202874000	-4.337241000	0.000064000
C	-4.055999000	-4.034736000	0.000454000
C	-2.732931000	-4.388350000	0.000474000
C	-1.706438000	-3.422010000	0.000184000
C	-2.072047000	-2.055201000	0.000174000
C	0.695535000	-2.804404000	-0.000028000
C	-0.309779000	-3.799871000	-0.000008000
C	0.088906000	-5.152111000	-0.000198000
C	1.409631000	-5.514366000	-0.000314000
O	3.818453000	9.141304000	-0.001034000
O	-9.887073000	-1.151831000	-0.001104000
O	7.899075000	-5.966889000	-0.000151000
H	1.288116000	9.552403000	-0.000954000
H	-0.452042000	7.838606000	-0.000616000
H	4.304219000	6.541393000	-0.000706000
H	-1.810707000	6.469319000	0.000022000
H	-3.493616000	4.784513000	0.000300000
H	5.376190000	2.380774000	0.000568000
H	4.773435000	4.685159000	0.000386000
H	-4.845376000	3.441776000	0.001989000
H	-6.540189000	1.765905000	0.001778000
H	6.481715000	-1.759999000	0.000752000
H	5.863710000	0.542867000	0.000833000
H	-7.929105000	0.428869000	-0.000111000
H	-8.998798000	-3.695458000	-0.001542000
H	-6.659001000	-4.333555000	-0.001049000
H	3.506643000	-7.084998000	-0.001121000
H	5.849107000	-7.714082000	-0.001031000
H	7.005623000	-3.613076000	0.000326000
H	-4.792865000	-4.826987000	0.000730000
H	-2.491181000	-5.442500000	0.000869000
H	-0.651829000	-5.940056000	-0.000384000
H	1.645985000	-6.570249000	-0.000362000
H	4.709785000	8.773462000	-0.000805000
H	-10.474288000	-1.916034000	-0.001023000
H	8.019442000	-6.923076000	-0.000462000

(b) first excited state

C	-6.598752000	-5.632606000	0.000137000
C	-6.228578000	-4.301715000	0.000169000
C	-4.874385000	-3.913880000	0.000113000
C	-3.867122000	-4.922738000	0.000023000
C	-4.282869000	-6.274973000	-0.000007000
C	-5.611822000	-6.633632000	0.000047000
C	-4.481019000	-2.516953000	0.000145000
C	-3.104414000	-2.154750000	0.000086000
C	-2.095499000	-3.170609000	-0.000004000
C	-2.472627000	-4.546208000	-0.000034000
C	-5.438945000	-1.484496000	0.000233000
C	-5.073320000	-0.161043000	0.000269000
C	-3.723641000	0.239232000	0.000223000
C	-2.719120000	-0.775415000	0.000128000
C	-0.715994000	-2.798161000	-0.000074000
C	0.291932000	-3.809094000	-0.000164000

C	-0.121632000	-5.162371000	-0.000180000
C	-1.443584000	-5.514472000	-0.000118000
C	-3.345842000	1.630253000	0.000273000
C	-1.967359000	1.995870000	0.000204000
C	-0.961528000	0.979358000	0.000107000
C	-1.340236000	-0.409909000	0.000052000
C	-0.338346000	-1.418419000	-0.000034000
C	1.047625000	-1.053852000	-0.000118000
C	2.054595000	-2.065648000	-0.000211000
C	1.676654000	-3.444071000	-0.000243000
C	-4.307617000	2.659139000	0.000389000
C	-3.950979000	3.985747000	0.000430000
C	-2.604504000	4.390749000	0.000362000
C	-1.599909000	3.378983000	0.000251000
C	3.439508000	-1.709968000	-0.000290000
C	4.444971000	-2.719459000	-0.000394000
C	4.027050000	-4.071581000	-0.000416000
C	2.703926000	-4.416545000	-0.000344000
C	0.410253000	1.340334000	0.000021000
C	1.422943000	0.320782000	-0.000068000
C	-2.236496000	5.795957000	0.000408000
C	-0.858131000	6.155029000	0.000336000
C	0.155510000	5.120856000	0.000213000
C	-0.216343000	3.742832000	0.000174000
C	3.816928000	-0.328618000	-0.000248000
C	5.193732000	0.032503000	-0.000311000
C	6.219978000	-0.986911000	-0.000436000
C	5.842152000	-2.360852000	-0.000475000
C	-3.207800000	6.815839000	0.000526000
C	-2.853554000	8.151784000	0.000574000
C	-1.497146000	8.514177000	0.000506000
C	-0.532333000	7.529828000	0.000389000
C	1.529548000	5.431497000	0.000126000
C	2.492862000	4.455466000	0.000019000
C	2.164396000	3.082113000	-0.000005000
C	0.784402000	2.722397000	0.000069000
C	2.802070000	0.681477000	-0.000146000
C	3.175408000	2.061741000	-0.000098000
C	4.550478000	2.377680000	-0.000143000
C	5.517800000	1.407602000	-0.000246000
C	6.872517000	-3.335653000	-0.000596000
C	8.203195000	-2.999581000	-0.000674000
C	8.570191000	-1.641109000	-0.000635000
C	7.596596000	-0.662887000	-0.000519000
O	9.904961000	-1.365389000	-0.000718000
O	-7.931754000	-5.922497000	0.000194000
O	-3.857998000	9.076127000	0.000691000
H	-7.027924000	-3.573735000	0.000240000
H	-3.548515000	-7.069325000	-0.000077000
H	-5.894539000	-7.683575000	0.000020000
H	-6.494759000	-1.719448000	0.000270000
H	-5.861621000	0.579334000	0.000334000
H	0.613210000	-5.955753000	-0.000241000
H	-1.686684000	-6.568770000	-0.000132000
H	-5.362499000	2.420820000	0.000450000
H	-4.742344000	4.723214000	0.000521000
H	4.758899000	-4.868055000	-0.000490000

H	2.456453000	-5.469397000	-0.000367000
H	-4.265854000	6.593005000	0.000583000
H	-1.207764000	9.562302000	0.000546000
H	0.503258000	7.843318000	0.000341000
H	1.856258000	6.463161000	0.000137000
H	3.528156000	4.768540000	-0.000050000
H	4.871851000	3.410504000	-0.000091000
H	6.552080000	1.725304000	-0.000267000
H	6.621525000	-4.387968000	-0.000633000
H	8.980375000	-3.755005000	-0.000767000
H	7.914933000	0.373712000	-0.000500000
H	10.041255000	-0.410650000	-0.000679000
H	-8.058526000	-6.877984000	0.000171000
H	-3.483533000	9.964222000	0.000709000

Supplementary Table 13. Optimized Cartesian coordinates (Å) of T-CQDs-COOH-3 for the ground state (a) and the first excited state (b).

(a) ground state

C	6.550499000	-5.675916000	0.008974000
C	6.167481000	-4.346631000	0.006310000
C	4.812787000	-3.962269000	0.004847000
C	3.820209000	-4.975673000	0.006314000
C	4.236250000	-6.328117000	0.008945000
C	5.566116000	-6.680758000	0.010256000
C	4.414103000	-2.562467000	0.002075000
C	3.043209000	-2.208244000	0.001687000
C	2.036336000	-3.235061000	0.003194000
C	2.415639000	-4.600782000	0.005184000
C	5.369851000	-1.528189000	-0.000418000
C	4.998544000	-0.209982000	-0.002677000
C	3.643365000	0.178369000	-0.002537000
C	2.653544000	-0.832277000	-0.000638000
C	0.653032000	-2.870683000	0.002747000
C	-0.339779000	-3.878825000	0.003293000
C	0.072536000	-5.227191000	0.005425000
C	1.396827000	-5.574164000	0.006430000
C	3.256123000	1.573037000	-0.004791000
C	1.886625000	1.928146000	-0.005558000
C	0.880497000	0.899068000	-0.002889000
C	1.260735000	-0.469845000	-0.000410000
C	0.264691000	-1.484491000	0.001462000
C	-1.110987000	-1.129376000	0.003033000
C	-2.121696000	-2.154291000	0.004271000
C	-1.741015000	-3.517195000	0.002597000
C	4.216916000	2.604700000	-0.005278000
C	3.855610000	3.925658000	-0.007857000
C	2.503309000	4.318232000	-0.011355000
C	1.511091000	3.308053000	-0.009303000
C	-3.508568000	-1.804123000	0.006994000
C	-4.501057000	-2.815683000	0.008340000
C	-4.081550000	-4.160494000	0.001608000
C	-2.754671000	-4.497475000	-0.000897000
C	-0.496137000	1.254342000	-0.001173000
C	-1.491397000	0.240560000	0.002084000
C	2.123224000	5.723109000	-0.019138000
C	0.749619000	6.077618000	-0.021740000
C	-0.261174000	5.033193000	-0.010583000
C	0.118504000	3.667869000	-0.008089000
C	-3.893018000	-0.418165000	0.006804000
C	-5.264140000	-0.064179000	0.007076000
C	-6.290588000	-1.095992000	0.013336000
C	-5.910967000	-2.462852000	0.015859000
C	3.085468000	6.751136000	-0.029481000
C	2.736207000	8.089839000	-0.019608000
C	1.371814000	8.435591000	-0.041465000
C	0.410961000	7.449673000	-0.045177000
C	-1.635650000	5.341869000	-0.000780000
C	-2.591181000	4.361354000	0.004692000
C	-2.249072000	2.993581000	0.001003000
C	-0.878421000	2.642182000	-0.002913000
C	-2.885683000	0.597104000	0.003980000
C	-3.262961000	1.960699000	0.002529000

C	-4.636900000	2.276538000	0.000753000
C	-5.600241000	1.303213000	0.002351000
C	-6.929773000	-3.442504000	0.035689000
C	-8.264074000	-3.103699000	0.029267000
C	-8.646636000	-1.749124000	0.008931000
C	-7.662091000	-0.777129000	0.021986000
C	-10.073853000	-1.291064000	0.021041000
O	-10.415601000	-0.187547000	0.349133000
O	-11.004910000	-2.202570000	-0.369889000
C	8.004219000	-5.980057000	0.010589000
O	8.888747000	-5.156608000	0.009862000
O	8.261504000	-7.310848000	0.013118000
C	3.846785000	9.096567000	-0.032679000
O	4.974451000	8.838798000	-0.355311000
O	3.521783000	10.360590000	0.350942000
H	6.960152000	-3.611669000	0.005520000
H	3.502039000	-7.122247000	0.010176000
H	5.858370000	-7.723040000	0.012335000
H	6.426273000	-1.759685000	-0.000771000
H	5.781717000	0.535306000	-0.004965000
H	-0.658821000	-6.023420000	0.007207000
H	1.645919000	-6.626564000	0.008314000
H	5.272255000	2.370041000	-0.003397000
H	4.641421000	4.668620000	-0.007503000
H	-4.808555000	-4.961287000	-0.003706000
H	-2.499796000	-5.548144000	-0.006255000
H	4.143210000	6.529578000	-0.048000000
H	1.051786000	9.471881000	-0.103781000
H	-0.626374000	7.753960000	-0.078769000
H	-1.966294000	6.371753000	0.005314000
H	-3.628435000	4.666306000	0.012880000
H	-4.962329000	3.307582000	-0.003041000
H	-6.636523000	1.612710000	-0.000293000
H	-6.674226000	-4.492903000	0.068258000
H	-9.001695000	-3.898979000	0.088515000
H	-7.999649000	0.249527000	0.041690000
H	-10.584459000	-2.982487000	-0.752649000
H	9.225381000	-7.408059000	0.014041000
H	2.634162000	10.388669000	0.728901000

(b) first excited state

C	-6.538494000	-5.693535000	-0.008112000
C	-6.154507000	-4.361827000	-0.005450000
C	-4.803297000	-3.974971000	-0.004248000
C	-3.800078000	-4.989757000	-0.006067000
C	-4.220343000	-6.346267000	-0.008622000
C	-5.548312000	-6.697407000	-0.009638000
C	-4.405654000	-2.577856000	-0.001471000
C	-3.030282000	-2.220121000	-0.001532000
C	-2.025510000	-3.240261000	-0.003106000
C	-2.409816000	-4.615568000	-0.005255000
C	-5.359676000	-1.542972000	0.001525000
C	-4.992428000	-0.218772000	0.003526000
C	-3.643734000	0.175374000	0.002621000
C	-2.642867000	-0.841516000	0.000747000
C	-0.648070000	-2.873307000	-0.002816000
C	0.359568000	-3.885714000	-0.003052000
C	-0.060209000	-5.241853000	-0.005877000
C	-1.380090000	-5.588902000	-0.007080000

C	-3.260318000	1.567891000	0.004268000
C	-1.884953000	1.927892000	0.004573000
C	-0.880489000	0.907314000	0.002556000
C	-1.262686000	-0.480042000	-0.000122000
C	-0.264337000	-1.493960000	-0.001225000
C	1.119337000	-1.133892000	-0.003156000
C	2.125791000	-2.149021000	-0.003519000
C	1.739729000	-3.526658000	-0.001390000
C	-4.218972000	2.597094000	0.004186000
C	-3.859854000	3.924727000	0.006189000
C	-2.513954000	4.322865000	0.009665000
C	-1.513204000	3.308480000	0.007936000
C	3.508051000	-1.797594000	-0.006341000
C	4.512574000	-2.809935000	-0.007224000
C	4.088423000	-4.163601000	0.001933000
C	2.767667000	-4.505118000	0.004309000
C	0.488670000	1.263937000	-0.000016000
C	1.497177000	0.241553000	-0.002311000
C	-2.137866000	5.728392000	0.017265000
C	-0.761089000	6.085440000	0.020051000
C	0.248936000	5.046019000	0.007739000
C	-0.128957000	3.669203000	0.005995000
C	3.888199000	-0.415911000	-0.006806000
C	5.263055000	-0.057877000	-0.007262000
C	6.289169000	-1.081431000	-0.013236000
C	5.906749000	-2.457929000	-0.016028000
C	-3.100495000	6.752951000	0.027338000
C	-2.754759000	8.095568000	0.018105000
C	-1.391291000	8.442980000	0.041910000
C	-0.427024000	7.458917000	0.045009000
C	1.621765000	5.353125000	-0.004318000
C	2.580406000	4.371420000	-0.009700000
C	2.244810000	3.001160000	-0.003604000
C	0.867704000	2.646614000	0.001327000
C	2.877804000	0.597973000	-0.004668000
C	3.255243000	1.976443000	-0.003738000
C	4.628608000	2.289497000	-0.001338000
C	5.592327000	1.314457000	-0.002530000
C	6.936701000	-3.434277000	-0.038165000
C	8.266261000	-3.089244000	-0.031942000
C	8.649427000	-1.729596000	-0.007391000
C	7.658939000	-0.761262000	-0.020073000
C	10.069385000	-1.265539000	-0.014299000
O	10.405879000	-0.147201000	-0.302017000
O	11.012051000	-2.186256000	0.332429000
C	-7.987119000	-5.998443000	-0.009420000
O	-8.875356000	-5.176300000	-0.008509000
O	-8.244889000	-7.331171000	-0.011938000
C	-3.868268000	9.096577000	0.032552000
O	-4.998076000	8.828859000	0.341677000
O	-3.547002000	10.367501000	-0.331726000
H	-6.949452000	-3.629052000	-0.004452000
H	-3.486434000	-7.140670000	-0.009935000
H	-5.839959000	-7.739959000	-0.011682000
H	-6.415735000	-1.775835000	0.002398000
H	-5.780303000	0.521582000	0.006267000
H	0.672226000	-6.037010000	-0.008500000

H	-1.628150000	-6.641474000	-0.009594000
H	-5.274302000	2.362301000	0.002231000
H	-4.649537000	4.663500000	0.005493000
H	4.818941000	-4.961084000	0.010170000
H	2.516862000	-5.556761000	0.012228000
H	-4.158079000	6.530164000	0.045149000
H	-1.072538000	9.479540000	0.105382000
H	0.609308000	7.766576000	0.079812000
H	1.952935000	6.382734000	-0.012634000
H	3.616730000	4.679396000	-0.020002000
H	4.956004000	3.319928000	0.002853000
H	6.628165000	1.625187000	0.000382000
H	6.686601000	-4.485944000	-0.074491000
H	9.005765000	-3.882716000	-0.094626000
H	7.995480000	0.265952000	-0.036341000
H	10.600078000	-2.981221000	0.692306000
H	-9.208835000	-7.425946000	-0.012655000
H	-2.657472000	10.405777000	-0.704137000

Supplementary Table 14. The calculated emission wavelength (nm), FWHM (nm), HOMO (eV), LUMO (eV) and band gap energies (eV) of the different kinds of model CQDs.

	S-CQDs			T-CQDs			T-CQDs-OH			T-CQDs-COOH		
	1	2	3	1	2	3	1	2	3	1	2	3
Em	398	496	524	312	411	481	323	430	491	354	434	500
FWHM	85	132	149	52	74	77	48	62	64	68	103	135
HOMO	5.39	5.16	5.06	5.96	5.49	5.27	5.34	5.25	5.07	6.85	6.30	5.65
LUMO	1.95	2.25	2.40	1.38	1.75	2.19	1.26	1.72	2.11	2.98	2.88	2.78
GAP	3.44	2.91	2.66	4.58	3.74	3.08	4.08	3.53	2.96	3.87	3.42	2.87

Supplementary Table 15. Calculated energy of different kinds of CQDs (Hartree, 1Hartree=2625.5 kJ/mol).

	1	2	3
S-CQDs	-615.9	-1228.2	-2300.4
T-CQDs	-693.3	-1383.1	-2301.5
T-CQDs-OH	-1144.8	-1608.8	-2527.3

Supplementary Table 16. Comparison of our high color-purity NBE-T-CQDs-based LEDs with other previously reported high-performance semiconductor QDs-based LEDs.

LEDs	EL / FWHM (nm)	V_{ON} (V)	L_{max} (cd m^{-2})	η_c (cd A^{-1})	Reference
B-LEDs	476 / 30	4.3	1882	1.22	*This work*
G-LEDs	510 / 32	3.7	4762	5.11	
Y-LEDs	540 / 38	3.5	2784	2.31	
R-LEDs	602 / 39	3.1	2344	1.73	
blue	470 / 28	2.4	4200	0.32	*1*
green	525 / 32	3-4	3700	1.1	*2*
yellow	576 / 35		4470	1.3	
orange	595 / 28		3200	1.8	
red	619 / 28		9064	2.8	
green	517 / N/A	N/A	154	0.3	*3*
blue	455 /20	5.1	742	0.14	*4*
green	516 / 23	4.2	946	0.43	
orange	586 / 23	4.6	528	0.08	
blue	490 / 19	3.0	35	N/A	*5*
green	515 / 19	3.0	330		
green	510 / N/A	N/A	2935	4.9	*6*
green	518 / 64	2.8	4182	1.53	*7*

Supplementary Table 17. Performance comparison of the NBE-T-CQDs-based LEDs fabricated with and without using PVK polymer host.

LEDs		L_{max} (cd m^{-2})	η_c (cd A^{-1})		L_{max} (cd m^{-2})	η_c (cd A^{-1})
B-LEDs	With PVK	1882	1.22	Without PVK	162	0.07
G-LEDs		4762	5.11		260	0.35
Y-LEDs		2784	2.31		126	0.09
R-LEDs		2344	1.73		92	0.06

Supplementary References

1. Qian, L. et al. Stable and efficient quantum-dot light-emitting diodes based on solution-processed multilayer structures. *Nat. Photonics* ***5***, 543-548 (2011).

2. Sun, Q. et al. Bright, multicoloured light-emitting diodes based on quantum dots. *Nat. Photonics* ***1***, 717-722 (2007).

3. Tan, Z. K. et al. Bright light-emitting diodes based on organometal halide perovskite. *Nat. Mater.**9***, 687-692 (2014).

4. Song, J. Z. et al. Quantum Dot Light-Emitting Diodes Based on Inorganic Perovskite Cesium Lead Halides ($CsPbX_3$). *Adv. Mater.* ***27***, 7162-7167 (2015).

5. Pan, J. et al. Highly Efficient Perovskite-Quantum-Dot Light-Emitting Diodes by Surface Engineering. *Adv. Mater.* ***28***, 8718-8725 (2016).

6. Byun, J. et al. Efficient Visible Quasi-2D Perovskite Light-Emitting Diodes. *Adv. Mater.* ***28**,* 7515-7520 (2016).

7. Lim, J. et al. Highly Efficient Cadmium-Free Quantum Dot Light-Emitting Diodes Enabled by the Direct Formation of Excitons within InP@ZnSeS Quantum Dots. *ACS Nano**7***, 9019-9026 (2013).
